# Research on the path of social psychological collaborative education in colleges and universities driven by the dynamic reward and punishment mechanism of the government

**DOI:** 10.1371/journal.pone.0340411

**Published:** 2026-02-19

**Authors:** Jingjia Guo, Yang Gao, Fangcheng Tang

**Affiliations:** Beijing University of Chemical Technology, Shanxi University of Finance and Economics, Beijing, China; USTC: University of Science and Technology of China, CHINA

## Abstract

This study addresses the prevalent issues of “coordination failure” and “cooperative inertia” in the collaborative education of mental health between universities and society. Utilizing evolutionary game theory, it systematically constructs and analyzes four models that combine government rewards and punishments. The findings indicate that while the traditional static reward and punishment mechanism offers basic incentives, its rigid design results in the system’s path dependence on government subsidies, thereby hindering the development of sustainable endogenous motivation. In order to address this governance dilemma, this paper first put forward the idea of combining “performance-based grant-reputation incentive” linkage system, this mechanism has prompted the external incentives to the internal incentives transformation effect under the joint governance of short-term fiscal policy and long-term reputation asset through the dynamic adjustment of incentive coefficient and reputation incentive resources are put into use. Numerical simulation results show that the “dynamic reward - static punishment” hybrid model of the two has the best policy effect. In the initial stage of cooperative relationship, government funding helps to break the situation of cooperation cannot be broken; In the later stage of the cooperation, the pursuit of reputation capital has become the motive for cooperating in deeper. It can be learned from relevant studies that reasonably allocating more incentive resources to universities that take the initiative can significantly enhance the efficiency of the system evolving into the state of deep cooperation and open sharing. Both theoretical and empirical evidence confirms that the proposed dynamic linkage mechanism markedly outperforms the traditional static model in terms of policy adaptability, incentive sustainability, and institutional robustness. This finding not only enriches the understanding of the evolutionary dynamics of the psychological healthy education system but also provides a theoretical foundation and practical pathway for establishing an incentivized, compatible, and sustainable governance system for mental health education.

## 1. Introduction

The global adolescent mental health crisis has emerged as a significant public health and educational governance challenge that demands urgent attention. The World Health Organization (WHO) highlighted in its 2022 World Mental Health Report that one in eight individuals worldwide experiences mental disorders, with mental health conditions being the primary contributor to disability among adolescents aged 10–19 in terms of disease burden (WHO, 2022) [1]. This trend is notably reflected in the United States, where data from the Centers for Disease Control and Prevention (CDC) in 2023 indicate that approximately 42% of high school students consistently feel sad or hopeless, and 22% of respondents have seriously contemplated suicide within the past year (CDC, 2023) [2]. Furthermore, the report from the United Nations Children’s Fund (UNICEF) underscores that mental health challenges have become the leading cause of dissatisfaction among European teenagers, posing a long-term threat to the social human capital reserves (UNICEF, 2021) [3].

In light of the ongoing crisis, establishing an effective “university-society” collaborative psychological education system has emerged as a critical task for ensuring the security of the national talent strategy. Globally, governments have increasingly allocated policy resources toward collaborative education initiatives. For instance, the United States designated over 380 million US dollars in fiscal year 2022 through federal programs such as the “School-based Mental Health Program”, which aims to integrate community psychological service resources into schools. Nevertheless, this initiative encounters the challenge of having resources that are difficult to integrate. Similarly, South Korea’s “Shared School Program” promotes community engagement in schools; however, collaboration often remains limited to individual activities, with societal motivation waning due to inconsistent returns. Furthermore, the “Special Action Plan for Comprehensively Strengthening and Improving Students’ Mental Health Work in the New Era (2023-2025),” jointly issued by 17 departments, including the Ministry of Education of China, emphasizes the need to “improve the collaborative education mechanism among schools, families, and society”. Yet, akin to the situations of other nations, a significant disparity persists between policy aspirations and actual outcomes.

In terms of theory, the collaboration of schools and communities can be viewed as an evolutionary game system under the regulation. In special occasions such as psychological crisis intervention and course co-creation, the cost-benefit structure contained in the strategic choice of universities and society, as boundedly rational actors, is significantly influenced. When one party chooses a strategy of “deep cooperation” or “resource openness”, it will bear a large amount of additional cost and risk, and the positive externalities brought about by cooperation are difficult to be fully monopolized. Under the structure of a static reward and punishment mechanism, such structural contradiction makes “remaining unchanged” and “adopting a conservative and wait-and-see attitude” the dominant strategy choices for individuals. As a result, the system is locked into a low-level equilibrium state with a collaborative inertia of “not moving unless the government pushes and not stopping unless the government stops.

Although there are already many research results that have laid some foundations for us to understand this situation, the existing literature tends to reduce the government’s intervention to a one-time subsidy or penalty. In addition, evolutionary games, which can be very useful for studying the interaction of the sides during the long process, also lack usage here. This relatively inadequate research on the dynamic mechanism design and evolutionary path analysis of the system has restricted the overall exploration of the approach to breaking the collaborative inertia.

This paper studies the Design of adaptive and sustainable dynamic incentive systems under the framework of government intervention, aiming to effectively drive the evolution of school-society collaboration toward a high-quality and stable state of “deep cooperation - open sharing”. To this end, an evolutionary game model under government intervention is constructed for both schools and society. This study first proposed that the essence of the dynamic linkage mechanism based on the relationship between “performance grants and reputation incentives” is the strategic transformation of the government’s role from the past “resource allocator” to the current “system ecosystem shaper”, which fundamentally reshaped the incentive structure of the game by building a closed-loop feedback system of “behavior - reputation - benefit”, which integrates short-term financial support and long-term reputation capital of the participants, and behavior incentive principles, such as target gradient effect and mental accounting. As a result, it leads the strategies of all parties to evolve spontaneously in a coordinated direction.

This study will examine two key issues through modeling analysis: 1. What are the theoretical advantages and the incentive compatibility logic underlying the dynamic linkage mechanism of “performance grant - reputation incentive”? How does this mechanism improve upon the static model? 2. Can this dynamic mechanism effectively promote the system’s evolution toward a high-quality equilibrium? Which hybrid model demonstrates the most effective policy outcomes?

## 2. Literature review and research orientation

### 2.1. Performance-based grants and reputation mechanism

In the pursuit of fostering collaborative partnerships between universities and society, performance-based grants and reputation mechanisms serve as two essential policy instruments. Research indicates that implementing a dynamic reward coefficient, which correlates with the investment in university cooperation and the openness of social services, along with linking financial resources to measurable performance outcomes, can effectively encourage universities to enhance their investment in specific initiatives [4]. The reputation mechanism, which is acknowledged for its ability to create long-term social capital [5], can encourage continuous cooperative behavior in a non-monetary form. In addition, based on research on the co-construction mechanism of mental health services for college students [6] and the study on mental health education strategies [7], it can be seen that in the collaborative educational mechanism for dealing with the mental health of college students, reputation incentives can be granted to participating universities through symbolic rewards to encourage cooperative behavior. In addition, ensure the system is able to bear the costs of keeping it working and some slight failures such as trusting the users is broken up over the long term.

Recent research identifies critical components for establishing an effective incentive system. In the context of progressive reputation dynamics, a multi-level and accumulative reputation system offers a more precise reflection of cooperation levels compared to a simple binary evaluation [8].In addition, it is necessary to keep maintenance costs and occasional acts of betrayal to maintain the long-term stability of the reputation system. All together now, it points out the good manner of incentives is variable, forgiving and bold. However, most of the existing literature is static interventions and is not effectively combined with these elements [9].

The “performance grant - reputation incentive” linkage mechanism proposed in this study is to create a dynamic mechanism with the above characteristics. Using multi-agent games theory and LLM simulation to verify, the results show that the effectiveness of reputation incentives has distinct stage dependence [10]. This observation is consistent with the role of the public sector as the initiative of the collaborative ecosystem [11]. The social efficiency deficit introduces a new dimension for quantifying the optimal collaboration and equilibrium gap [12]. Incentive design highlights the importance of commitment to participation, and it is also a guarantee for the completion, which enhances the adaptability of the dynamic mechanisms [13]. Different from the simple coercion is the coordination of collaborative behavior more conducive to the stability of cooperative relationship [14]. The static structural causes of the problems of dual-strategy games also provide a theoretical basis for dynamic mechanisms to break through the defects of weak advantage strategies [15]. Change short term fiscal incentive to long reputation capital to break synergy dilemma.

### 2.2. Research on the application of evolutionary game theory in educational collaboration

Evolutionary game theory serves as a critical analytical framework for examining the dynamic processes involved in collaborative education between schools and communities. Its primary value lies in challenging the traditional assumption of complete rationality, thereby offering a more accurate explanation of the evolutionary dynamics of group strategies under conditions of finite rationality [16]. This theoretical framework is more applicable to the analysis of the interaction and adjustment of strategies among all the stakeholders in the educational collaborative system. Research on how commitment and punishment interact makes it clear that cooperation evolution is shaped by those institutions that influence each other [17]. Social-physical mechanisms of group behavior evolution provide a new insight into collaborative modeling [18]. And, spatial reciprocal cluster expansion mechanism gives a new explanation for stability of the cooperation [19]. The development of structural complexity of the subunit self-organizing structure provides a micro-level reference for the orderly construction of collaborative systems [20]. Finally, the phase plane scale analysis of the intensity of social predicaments provides a quantification means to shift cooperative game type [21], the cooperative game together forms a methodological basis of the study long-term cooperation.

In the domain of educational collaborative research, the application of evolutionary game theory has revealed substantial theoretical significance. The notion of an evolutionary stable strategy serves as a crucial instrument for examining the long-term stability of strategies within collaborative systems [22]. Improve analytical methods for the dynamic evolution process, so that researchers can more precisely capture the long-term pattern of group behavior [23]. The establishment of a replicator dynamic model is an important technical path for studying the diffusion of strategies [24]. Studies of multi-person group games show that the more strategies there are and the more often they interact, the more susceptible they are to changing the equilibrium [25], which can help us understand the multiple agents involved in school-community collaboration strategy interaction is very complicated.

Evolutionary game theory and the combination with government intervention policies is just starting to emerge. The evolutionary game process constrained by institutional factors provides a new path for measuring the effectiveness of government reward and punishment mechanisms [26]. It is indicated by the research that the material restrictions can be applied to the subject of inaction [27]. The design of this punishment mechanism can effectively enhance cooperation and the function of the system by increasing the cost of non-cooperation, and emphasize the role of reputation in promoting long-term cooperation [28]. These developments, in turn, further advance the theory of collaborative governance within the field of education.

Based on these theories, a dynamic evolution game model will be developed in this study to explore the interaction mechanism among universities, the government and society under the change of variable rewards and punishments policy by the government. Through the systematic analysis of the evolution paths generated by different combinations of these incentives, provide theoretical support for the improvement of collaborative governance policy.

### 2.3. Research on cost-benefit structure and behavioral incentives in collaborative cooperation

Sustainable development of school-community collaborative education needs to be based on comprehensive cost-benefit analysis [29]. Some related studies have systematizedly elaborated on the revenue system of the school-society collaborative model, and it is clearly stated therein that the revenue from the school-society collaboration covers value creation from resource complementarity, professional collaboration, and scale effects, etc. This system of revenue is usually a means to reflect the capacity to expand the scope of mental health services and the effect of carrying out the intervention through joint activities. In addition, the collaborative revenue of society evaluates the degree to which it can benefit from the overall improvement of the public’s psychological literacy and the strengthening of the psychological crisis prevention network through the collaborative mechanism. This research provides a reference for the overall value orientation of the study of collaborative education.

Cost classification framework of cost structure analysis consist mainly management coordination, human resources and operation and maintenance. According to an extended study of the same study: the marginal investment required to move from closed maintenance to open collaboration is an important economic factor affecting the improvement of greater educational effect and efficiency. Pre-commitment mechanism can increase the efficiency of collaborative coordination in asymmetric revenue context significantly with benefit sharing clarify and responsibility defining. The subsidy matching logic contained in asymmetric games can optimize the cost-benefit structure of collaboration [30], and the evolutionary dynamics of behavioral strategies in public-private partnerships can be referred to in school-community collaboration [31]. The loss aversion characteristic identified by prospect theory [32] provides a key understanding of the risk-averse behavior of the participants in the collaborative model. Moreover, the target gradient effect is able to explain the difference in input intensity among participants when they move closer to their goals [33].

In Designing incentive mechanisms, mental accounting theory shows that people treat incentives from different sources differently [34]. Fairness preferences and incentive mechanisms [35] have increased our knowledge of the behavioral intents of the collaborative agent. The collective theoretical achievements form the basis of knowledge for the Design of incentive mechanisms.

In terms of the loss mechanism, the closed nature of the social resource system hasan adverse effect on mental health education in institutions of higher education and directly reduces the effectiveness of such education. This phenomenon is particularly reflected in the reduction of educational capacity of institutions caused by the lack of external resources. Research on the reputation of research-oriented universities shows that these institutions suffer significant harm to their social reputation at the same time, and also lose cooperation opportunities [36]. Furthermore, there is a cost on the social dimension due to this closed strategy. In terms of missed key development opportunities caused by lack of effective collaboration with other universities, according to the opportunity cost logic analyzed in innovation research, this cost form According to the basic definition of the opportunity cost logic framework analyzed in innovation studies [37], when applying such cost framework to analyze university organizations, specific contexts need to be considered. This inevitably results in a slowdown in the improvement of professional service levels and a gradual loss of influence, as well as other long-term hazards. Based on the above discussion, this paper will adopt a cost-benefit analysis method to combine the key contents of behavioral incentive theory, and build a dynamic incentive mechanism that fits the behavioral characteristics of the subjects. Improve the stability and sustainability of the collaborative system.

To sum up, although some achievements have been made in the existing research on school-community collaboration in terms of performance-based grants and reputation mechanisms, the research perspectives are still lacking. First, many studies reduce the reduction of government intervention to a static model, ignoring the fact that the collaboration between schools and communities is an interactive dynamic process of continuous learning and strategic adaptation. Second, most research methods are based on comparative static analysis, lacking the use of evolutionary game theory, which is more suitable for studying the dynamic process. This gap is more pronounced in the in-depth exploration of the coordination mechanisms among universities, enterprises and institutions, and communities in response to the changing government rewards and punishments. In light of the above, this study will establish a school-society collaborative evolution game model based on the linkage mechanism of “performance grant - reputation incentive”, explore how effective institutional Design can motivate everyone to participate, and provide a new thinking path to break through the predicament of collaboration.

## 3. Evolution game analysis of government static reward and punishment mechanisms

**Fig 1:**
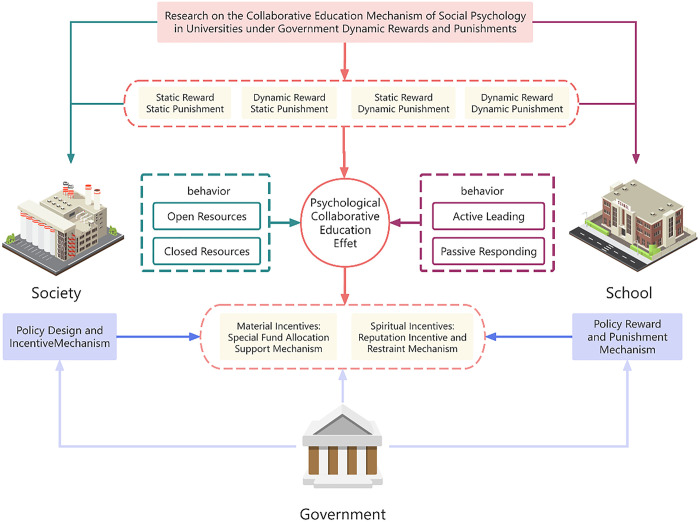
Schematic diagram ofschool-community collaboration mechanism under government incentives and sanctions.

In the absence of government incentives or punishment, the construction of a stable and effective school-community psychological education collaborative system is difficult to achieve, and it is in a low-level equilibrium state of “maintaining the status quo - resource isolation”. To break this equilibrium, a capable and reliable government with the ability to allocate resources must be introduced as an external intervenor. Using the incentive & punishment method properly, the way that changes the income between universities and social institutions involved in collaborative education can be adjusted, influencing their decision making. Fig 1 shows the main interaction process of the government, universities and society in the collaborative mental health education system, revealing the basic framework and parameter relationship of the game model developed in this paper. Therefore, Against the backdrop of the government’s static reward and punishment mechanism, this chapter develops an evolutionary game model and analyzes the impacts of policy instruments including fixed-sum fiscal subsidies, reputation incentives and economic penalties on the willingness of both parties to cooperate. Through a detailed examination of the game’s equilibrium status as well as the conditions for maintaining stability with this mechanism, This study is trying to find a theory on the minimal logic and effectiveness boundary of public intervention strategies.

### 3.1. Basic assumptions and model construction

In the model, the government adheres to the principle of practical feasibility and consists of two regulatory types: material and reputational. Material regulation involves controlling fiscal resources by recovering part or all of project grants from entities that fail to meet their collaborative commitments or by revoking their tax preference qualifications, rather than imposing direct fines. Reputational regulation influences the social image and long-term development capital of entities through actions like granting or revoking honorary titles and issuing public notifications.

**Hypothesis 1**: Under the under the government’s static reward and punishment mechanism, universities have a strategic space of {cooperation, maintenance}, while the social strategic space is {openness, conservatism}. The probability that universities choose “cooperation” is x (0 ≤ x ≤ 1), and the probability that society opts for “openness” is y (0 ≤ y ≤ 1).

**Hypothesis 2** (Revenue Hypothesis): The revenue model for school-community collaboration comprises direct revenue and total revenue from the partnership. When society adopts the “open” strategy, it receives direct benefits D and provides additional benefits E to universities. If both parties choose to collaborative strategy, a total collaborative benefit R^e^ is generated for universities and society. This total benefit is distributed according to the university’s income distribution ratio ξ, where universities receive ξR^e^ and society receives (1- ξ)R^e^. This distribution reflects the principles of value co-creation and risk sharing in collaborative education (Perkmann, et al.,2013;Bogers et al., 2017).

**Hypothesis 3** (Cost Hypothesis): In terms of cost structure, the universities adopting the “cooperative” strategy will face increased costs. Similarly, social organizations choosing an “open” strategy will incur additional costs denoted by cg. Conversely, if social organizations opt for a “conservative” approach, they will only bear the basic costs of closed operations. (Mora-Valentin et al., 2004)

**Hypothesis 4** (Loss Hypothesis): When society takes a conservative strategy, universities will experience both financial loss G2 and reputation loss L𝑏. Societally we must take into account additional damages M due to lost development caused by isolation.(Feller et al.,2002; Laursen and Salter et al. (2006).)

**Hypothesis 5** (Reputation Incentive Hypothesis): The government promotes the occurrence of collaborative behavior through the reputation incentive mechanism. Universities that adopt cooperative strategies and social groups that adopt open strategies should receive reputation incentives (Liang, 2021; Gong, 2020; Wang, 2023) ${\text{P}}_{\text{s}}$.

**Hypothesis 6** (constraint mechanism hypothesis): The government creates constraint mechanisms for regulating how the public participates. If the university is engaged in maintenance strategy, it should be subject to punishment, while if the social group is engaged in conservative strategy, it should incur both material and reputational penalties ${\text{F}}_{\text{1}}{\text{F}}_{\text{2}}$.

**Hypothesis 7** (Support Mechanism Hypothesis): The government promotes the development of collaborative systems by providing financial support. Specifically, the specific funds for mental health education are, and the resource distribution follows dynamic incentive coefficients and (Rutherford & Rabovsky, 2022; Li & Wu, 2023) Sζλ.

The following Table 1 gives the key game variables in the cooperation between universities and social forces for mental health education. This table covers the main contents such as the cost, benefits, loss of the strategic choice of both sides, and government reward and punishment, providing a quantitative basis for analyzing the balance of multi-party interaction.

**Table 1:** Model parameters and descriptions.

Parameter	Parameter Meaning	Range
${\text{C}}_{\text{A}}^{\text{S}}$	Additional Costs for Universities to Adopt a “Collaborative” Strategy in Joint Mental Health Education	${\text{C}}_{\text{A}}^{\text{S}}>\text{0}$
${\text{R}}^{\text{e}}$	Total Benefits of University-Community Mental Health Education Collaboration	${\text{R}}^{\text{e}}>\text{0}$
${\text{G}}_{\text{2}}$	Social losses incurred by universities when society chooses the “conservative” strategy	${\text{G}}_{\text{2}}>\text{0}$
${\text{C}}_{\text{O}}^{\text{C}}$	Additional Costs of Society Choosing an “Open” Strategy	${\text{C}}_{\text{O}}^{\text{C}}>\text{0}$
D	Direct Benefits to Society from Opening Mental Health Services	D>0
ξ	Proportion of Benefits Allocated to Universities	0≤ξ≤1
${\text{L}}_{\text{b}}$	Benchmark for reputational loss to universities, i.e., negative impact on institutional social standing and future collaboration opportunities	${\text{L}}_{\text{b}}>\text{0}$
M	Additional losses borne by society due to conservatism (e.g., diminished talent quality)	M>0
E	Additional benefits to universities from reduced pressure and improved governance efficiency due to societal openness	E>0
S	Government funding baseline (hereafter referred to as government funding)	S>0
${\text{P}}_{\text{c}}$	Government reputation incentives for university cooperation	${\text{P}}_{\text{1}}>\text{0}$
${\text{P}}_{\text{s}}$	Government reputation incentives for societal openness	${\text{P}}_{\text{2}}>\text{0}$
W	Government penalties for universities maintaining conservatism	W>0
${\text{F}}_{\text{1}}$	Government material penalties for social conservatism	${\text{F}}_{\text{1}}>\text{0}$
${\text{F}}_{\text{2}}$	Government reputational penalties for social conservatism	${\text{F}}_{\text{2}}>\text{0}$
ζ	Government Dynamic Incentive Intensity Coefficient for Universities	0≤ζ≤1
λ	Government Dynamic Incentive Intensity Coefficient for Society	0≤λ≤1
y	Probability of Society Choosing Openness	0≤y≤1
x	Probability of universities choosing cooperation	0≤x≤1

The payoff matrix for universities and society under government intervention is shown in the Table 2 below:

**Table 2:** Game theory payoffmatrix for universities and society.

	Society: Open Support (y)	Society:Conservative wait-and-see (1-y)
University: Deep Cooperation (x)	$\xi {\text{R}}^{\text{e}}-{\text{C}}_{\text{A}}^{\text{S}}+\text{E}+\zeta \text{S}+{\text{P}}_{\text{c}}$ $\left(\text{1}-\xi \right){\text{R}}^{\text{e}}-{\text{C}}_{\text{O}}^{\text{C}}+\text{D}+\lambda \text{S}+{\text{P}}_{\text{s}}$	$-{\text{G}}_{\text{2}}-{\text{C}}_{\text{A}}^{\text{S}}+\zeta \text{S}+{\text{P}}_{\text{c}}$ $-\text{M}-{\text{F}}_{\text{1}}-{\text{F}}_{\text{2}}$
University: Maintain the status quo (1-x)	$\text{E}-{\text{L}}_{\text{b}}-\text{W}$ $\text{D}-{\text{C}}_{\text{O}}^{\text{C}}+\lambda \text{S}+{\text{P}}_{\text{s}}$	$-{\text{L}}_{\text{b}}-{\text{G}}_{\text{2}}-\text{W}$ $-\text{M}-{\text{F}}_{\text{1}}-{\text{F}}_{\text{2}}$

### 3.2. Game analysis of the evolution of reward and punishment mechanisms between schools and societies under government intervention

#### 3.2.1. Equilibrium point assumption in the evolution process.

Within the group of universities, the proportion opting for the “deep cooperation” strategy is denoted as x (0 ≤ x ≤ 1), while those choosing the “maintain the status quo” strategy make up 1 – x. Similarly, in the group of social organizations, the proportion selecting the “open support” strategy is y (0 ≤ y ≤ 1), and those adopting the “conservative wait-and-see” strategy account for 1 – y.

For colleges and universities, the expected returns from the “cooperation” strategy, the “maintenance” strategy, and the average expected returns are defined as follows:


$$\pi_{x}=y(E-C_{A}^{S}+R^{e}\xi +\zeta S+P_{c})+(y-\text{1})(C_{A}^{S}+G_{\text{2}}-\zeta S-P_{c})$$

$$\pi_{\text{1}-x}=y(E-L_{b}-W)+(y-\text{1})(G_{\text{2}}+L_{b}+W)$$

$${\overset{―}{\pi }}_{x}=x\pi_{x}+(\text{1}-x)\pi_{\text{1}-x}$$

For society, the anticipated returns for selecting an “open” strategy, a “conservative” strategy, and the average expected return are as follows:


$$\pi_{\gamma }=x(D-C_{O}^{C}+\lambda S+P_{s}-R^{e}(\xi -\text{1}))-(x-\text{1})(D-C_{O}^{C}+\lambda S+P_{s})$$

$$\pi_{\text{1}-\gamma }=(x-\text{1})(F_{\text{1}}+F_{\text{2}}+M)-x(F_{\text{1}}+F_{\text{2}}+M)$$

$${\pi ¯}_{\gamma }=y\pi_{\gamma }+(\text{1}-y)\pi_{\text{1}-\gamma }$$

According to the Malthusian equation, the growth rate of “cooperative” strategies selected by universities is determined by the difference between their fitness and the average fitness. The replication dynamic equation is organized as follows:


$$F(x)=\frac{dx}{dt}=-x(x-\text{1})(L_{b}-C_{A}^{S}+W+P_{c}+S\zeta +R^{e}\xi y)$$

The replication dynamic equation for society is defined:


$$\text{F}(\text{y})=\frac{dy}{dt}=-y(y-\text{1})(D-C_{O}^{C}+F_{\text{1}}+F_{\text{2}}+M+P_{s}+S\lambda +R^{e}x-R^{e}x\xi )$$

with its equilibrium point being (0,0), (0,1), (1,0), (1,1), $\left(x^{*},y^{*}\right)$. When the condition is satisfied:


$$L_{b}-C_{A}^{S}+W+P_{c}+S\zeta <\text{0}$$

$$L_{b}-C_{A}^{S}+W+P_{c}+S\zeta +R^{e}\xi >\text{0}$$

$$D-C_{O}^{C}+F_{\text{1}}+F_{\text{2}}+M+P_{s}+S\lambda <\text{0}$$

$$D-C_{O}^{C}+F_{\text{1}}+F_{\text{2}}+M+P_{s}+S\lambda +R^{e}(\text{1}-\xi )>\text{0}$$

$\left(x^{*},y^{*}\right)$ this point also serves as the system’s equilibrium, $x^{*}=\frac{D-C_{O}^{C}+F_{\text{1}}+F_{\text{2}}+M+P_{s}+S\lambda }{R^{e}(\text{1}-\xi )},{\;y}^{*}=\frac{C_{A}^{S}-L_{b}-W-P_{c}-S\zeta }{R^{e}\xi }$.

Proof: For this system, let $\frac{dx}{dt}=\text{0},\frac{dy}{dt}=\text{0}$ and solve the replicator dynamics equation:


$$x(\text{1}-x)\left[L_{b}-C_{A}^{S}+W+P_{c}+S\zeta +R^{e}\xi y\right]=\text{0}$$

$$y(\text{1}-y)[D-C_{O}^{C}+F_{\text{1}}+F_{\text{2}}+M+P_{s}+S\lambda +R^{e}x(\text{1}-\xi )]=\text{0}$$

Clearly, (0,0), (0,1), (1,0), (1,1) are equilibrium points of the system.

When the conditions are satisfied, we have $\text{0}<\frac{D-C_{O}^{C}+F_{\text{1}}+F_{\text{2}}+M+P_{s}+S\lambda }{R^{e}(\text{1}-\xi )}<\text{1},\;\text{0}<\frac{C_{A}^{S}-L_{b}-W-P_{c}-S\zeta }{R^{e}\xi }<\text{1}$. Therefore, $\left(x^{*},y^{*}\right)$ are also equilibrium points of the system. Q.E.D.

#### 3.2.2 Equilibrium Point Stability Analysis.

The partial derivatives with respect to x and y are calculated for the replication dynamics equations of universities and social groups, respectively, yielding the system’s Jacobian matrix. Since the equilibrium points of the replication dynamics equations are asymptotically stable, they represent evolutionarily stable strategies. Based on the local stability analysis method for the Jacobian matrix (see S1 Appendix), stability analysis is conducted for the five equilibrium points. The specific results are presented in Table 3.

**Table 3:** Characterization of evolutionary stability in social and school game systems.

Equilibrium Point	Tr(J)	Det(J)
(0,0)	$(L_{b}-C_{A}^{S}+W+P_{c}+S\zeta +$ $\left(D-C_{O}^{C}+F_{\text{1}}+F_{\text{2}}+M+P_{s}+S\lambda \right)$	($L_{b}-C_{A}^{S}+W+P_{c}+S\zeta \;$) ($D-C_{O}^{C}+F_{\text{1}}+F_{\text{2}}+M+P_{s}+S\lambda$)
(0,1)	($C_{A}^{S}-L_{b}-W-P_{c}-S\zeta$) + ($D-C_{O}^{C}+F_{\text{1}}+F_{\text{2}}+M+R^{e}+P_{s}+S\lambda -R^{e}\xi$)	($C_{A}^{S}-L_{b}-W-P_{c}-S\zeta$) ($D-C_{O}^{C}+F_{\text{1}}+F_{\text{2}}+M+R^{e}+P_{s}+S\lambda -R^{e}\xi$)
(1,0)	($L_{b}-C_{A}^{S}+W+P_{c}+R^{e}\xi +S\zeta$)+($C_{O}^{C}-D-F_{\text{1}}-F_{\text{2}}-M-P_{s}-S\lambda$).	($L_{b}-C_{A}^{S}+W+P_{c}+R^{e}\xi +S\zeta$) ($\begin{array}{c} C_{O}^{C}-D-F_{\text{1}}-F_{\text{2}}-M-P_{s}-S\lambda \end{array}$)
(1,1)	($C_{A}^{S}-L_{b}-W-P_{c}-R^{e}\xi -S\zeta )$ +($C_{O}^{C}-D-F_{\text{1}}-F_{\text{2}}-M-R^{e}-P_{s}-S\lambda +R^{e}\xi$)	($C_{A}^{S}-L_{b}-W-P_{c}-R^{e}\xi -S\zeta$) ($C_{O}^{C}-D-F_{\text{1}}-F_{\text{2}}-M-R^{e}-P_{s}-S\lambda +R^{e}\xi$)
x*,y*	0	+

Changes in key parameters, such as the cost coefficient of university cooperation, the cost coefficient of social group openness, and the intensity of government rewards and punishments, significantly impact the system’s evolutionary stability strategy. Depending on the combinations of these parameter values, the system exhibits a variety of evolutionary paths and stable states.

(1) When $L_{b}-C_{A}^{S}+W+P_{c}+S\zeta <\text{0},\;L_{b}-C_{A}^{S}+W+P_{c}+S\zeta +R^{e}\xi >\text{0},\;D-C_{O}^{C}+F_{\text{1}}+F_{\text{2}}+M+P_{s}+S\lambda <\text{0},\;D-C_{O}^{C}$$+\;F_{\text{1}}+F_{\text{2}}+M+P_{s}+S\lambda +R^{e}(\text{1}-\xi )>\text{0}$, the system possesses two evolutionarily stable strategies (ESS): (maintain, conservative) and (cooperate, open).
(2) When ${\text{L}}_{\text{b}}-{\text{C}}_{\text{A}}^{\text{S}}+\text{W}+{\text{P}}_{\text{c}}+S\zeta <\text{0},{\text{L}}_{\text{b}}-{\text{C}}_{\text{A}}^{\text{S}}+\text{W}+{\text{P}}_{\text{c}}+S\zeta +{\text{R}}^{\text{e}}\xi >\text{0},\text{D}-{\text{C}}_{\text{O}}^{\text{C}}+{\text{F}}_{\text{1}}+{\text{F}}_{\text{2}}+\text{M}+{\text{P}}_{\text{s}}+S\lambda +{\text{R}}^{\text{e}}(\text{1}-\xi )<\text{0},$ the system’s evolutionarily stable strategy (ESS) is (maintain, conservative).
(3) When ${\text{L}}_{\text{b}}-{\text{C}}_{\text{A}}^{\text{S}}+\text{W}+{\text{P}}_{\text{c}}+S\zeta +{\text{R}}^{\text{e}}\xi <\text{0},\text{D}-{\text{C}}_{\text{O}}^{\text{C}}+{\text{F}}_{\text{1}}+{\text{F}}_{\text{2}}+\text{M}+{\text{P}}_{\text{s}}+S\lambda <\text{0},\text{D}-{\text{C}}_{\text{O}}^{\text{C}}+{\text{F}}_{\text{1}}+{\text{F}}_{\text{2}}+\text{M}+{\text{P}}_{\text{s}}+S\lambda$$+\;{\text{R}}^{\text{e}}(\text{1}-\xi )>\text{0},$ the system’s evolutionary stable strategy (ESS) is (maintain, conservative).
(4) When $\;L_{b}-C_{A}^{S}+W+P_{c}+S\zeta +R^{e}\xi >\text{0},\;D-C_{O}^{C}+F_{\text{1}}+F_{\text{2}}+M+P_{s}+S\lambda +R^{e}(\text{1}-\xi )<\text{0}$, the system’s evolutionary stable strategy (ESS) is (maintain, conservative).

Proof: We can examine the local stability of the five equilibrium points under different constraints by adopting the local stability analysis method based on the above Jacobian matrix.The findings of such analyses under various circumstances are presented in Tables 4–7.

**Table 4:** Local stability of the equilibrium point in case (1).

Equilibrium Point	Sign of Tr(J)	Det(J) Sign	Result
(0,0)	–	+	ESS
(0,1)	+	+	Unstable
(1,0)	+	+	Unstable
(1,1)	–	+	ESS

**Table 5:** Local stability of the equilibrium point in case (2).

Equilibrium point	Tr(J) Sign	Det(J) Sign	Result
(0,0)	–	+	ESS
(0,1)	Uncertain	–	Saddle point
(1,0)	+	+	Unstable
(1,1)	Uncertain	–	Saddle point

**Table 6:** Local stability of the equilibrium point in case (3).

Equilibrium Point	Tr(J) Symbol	Det(J) Sign	Result
(0,0)	–	+	ESS
(0,1)	+	+	Unstable
(1,0)	Uncertain	–	Saddle point
(1,1)	Uncertain	–	Saddle Point

**Table 7:** Local Stability of the Equilibrium Point in Case (4).

Equilibrium point	Tr(J) sign	Det(J) Sign	Result
(0,0)	–	+	ESS
(0,1)	Uncertain	+	Saddle point
(1,0)	Uncertain	+	Saddle point
(1,1)	+	+	Unstable

#### 3.2.3 Analysis of evolutionary outcomes.

**Fig 2:**
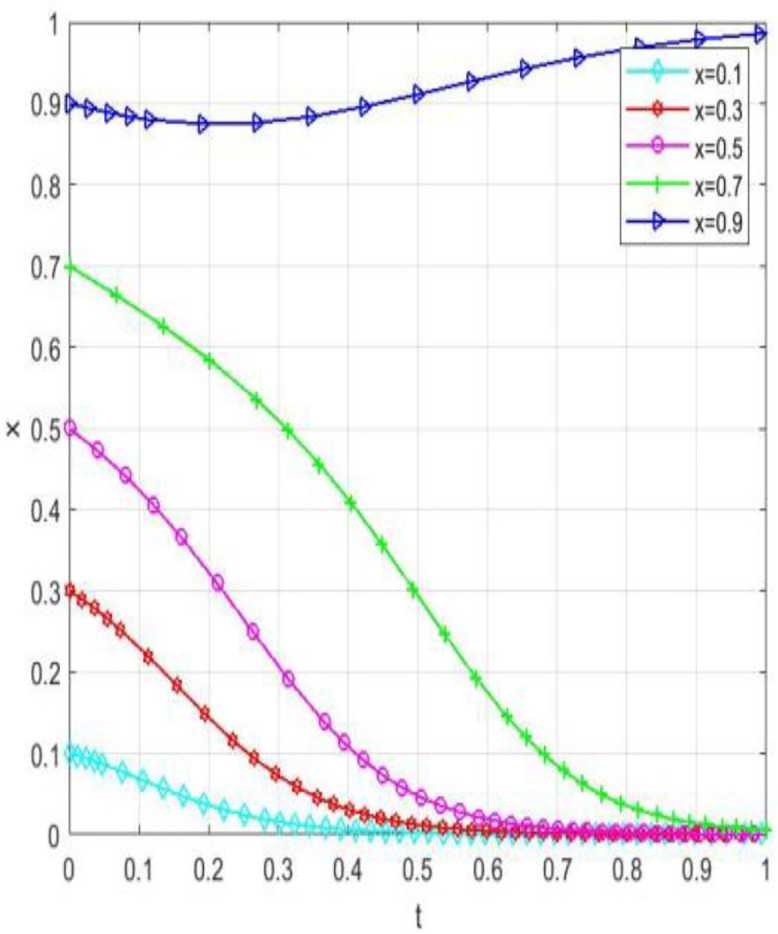
The impact of initial conditions in higher education institutions on evolutionary pathways.

**Fig 3:**
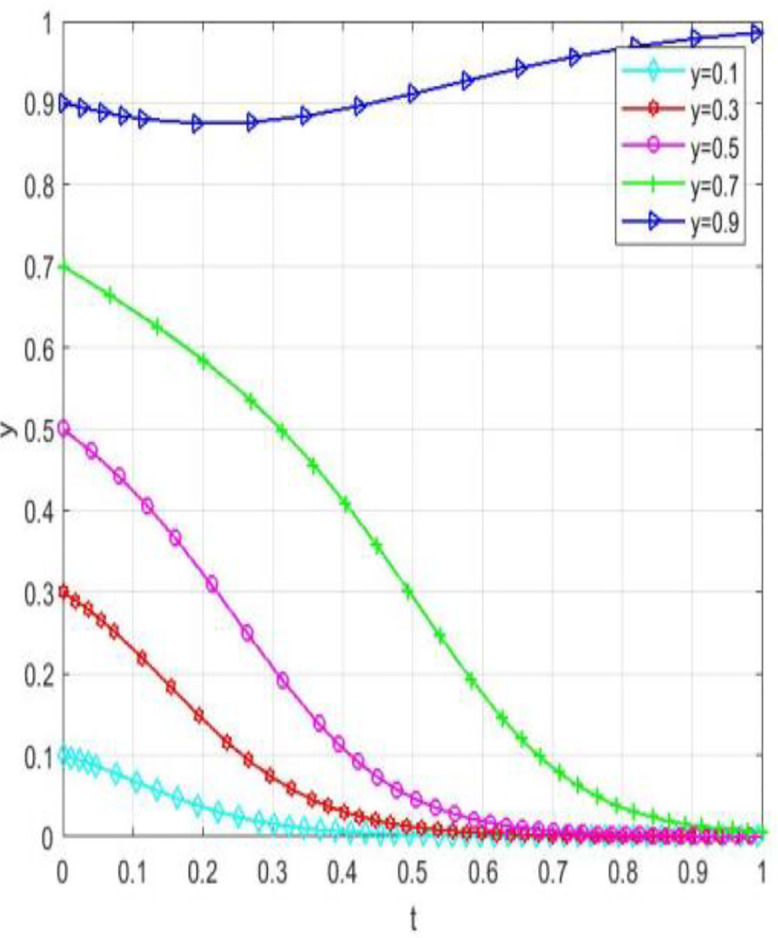
The impact of initial conditions on evolutionary trajectories in society.

**Fig 4:**
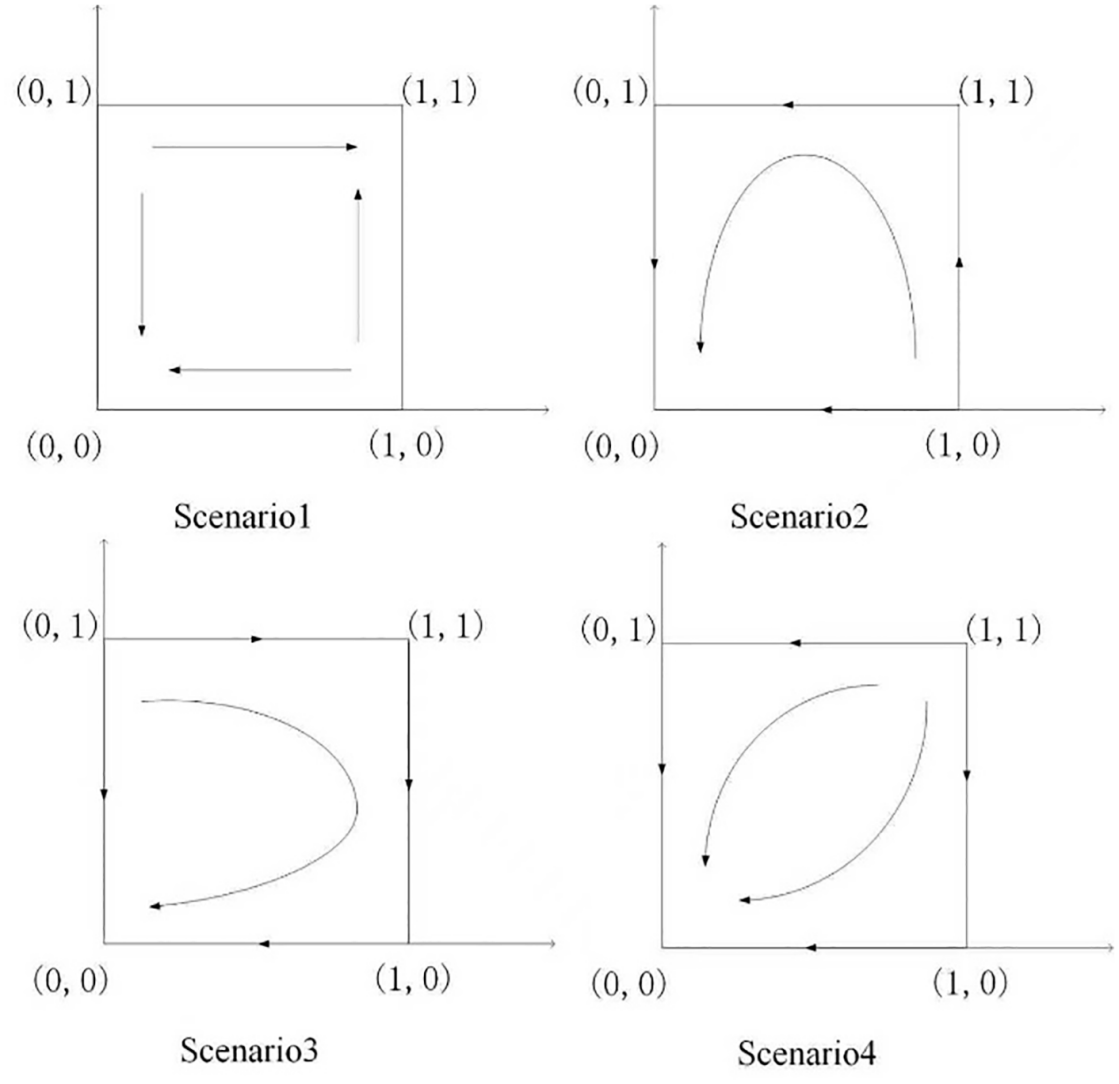
The impact of initial values in higher education on evolutionary.

**Fig 5:**
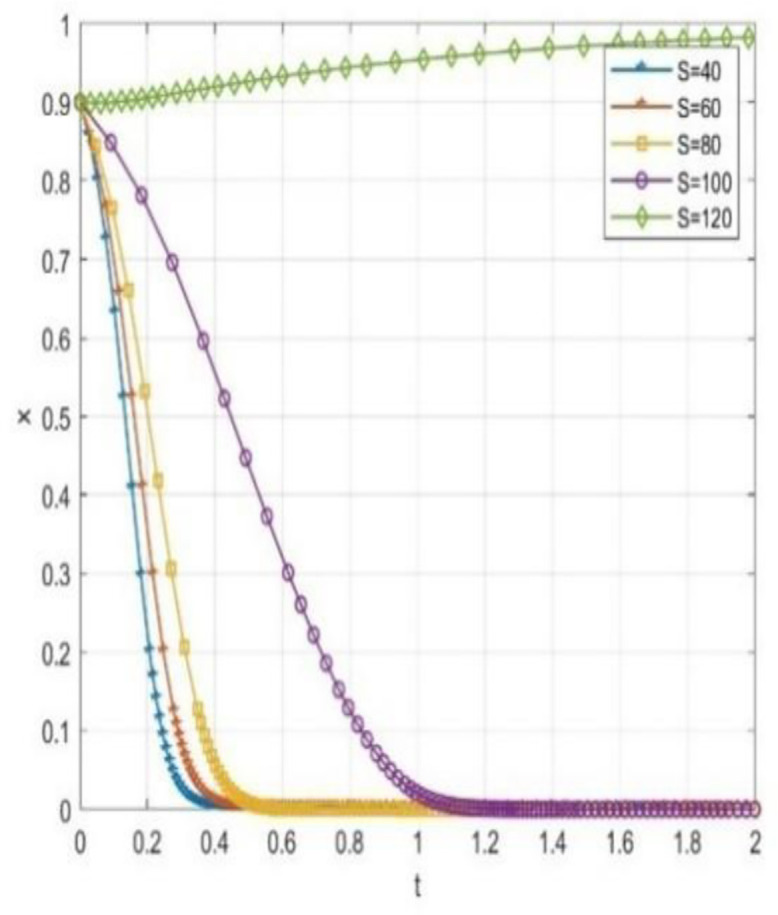
The impact of government funding on the probability of universities taking the Initiative.

Through numerical simulation, Fig 2 illustrates the impact of the initial will of colleges and universities on the evolutionary outcome, assuming that the parameter settings and the initial social will are held constant. Conversely, Fig 3 demonstrates the effect of the initial will of society on the evolutionary result, with the parameter settings and the initial will of universities remaining unchanged. Based on the analysis of Proposition 2,the evolutionarily stable strategies for universities and societal groups under different parameter conditions are shown in Fig 4 Specific analysis follows:

(1) When the benchmark values for net cooperative benefits in both higher education institutions and societal groups are negative, i.e., $L_{b}-C_{A}^{S}+W+P_{c}+S\zeta <\text{0}$ and $D-C_{\text{0}}^{C}+F_{\text{1}}+F_{\text{2}}+M+P_{s}+S\lambda <\text{0}$, but the cooperative payoff is sufficiently large that when the total payoff turns positive, the system exhibitsbistable characteristics. As shown in Fig 5. 1(a), both (0,0) and (1,1) are evolutionarily stable points, while (0,1) and (1,0) are saddle points. This indicates the system may converge to either “complete non-cooperation” or “full cooperation,” with the specific evolutionary path determined by the initial state.
(2) When the benchmark net benefit of university cooperation is negative but the total benefit is positive, while the total benefit of social group openness is negative,i.e., $L_{b}-C_{A}^{S}+W+P_{c}+S\zeta <\text{0}$, $L_{b}-C_{A}^{S}+W+P_{c}+S\zeta +R^{e}\xi >\text{0}$, $D-C_{\text{0}}^{C}+F_{\text{1}}+F_{\text{2}}+M+P_{s}+S\lambda +R^{e}(\text{1}-\xi )<\text{0}$, as shown in Fig 5. 1(b). Here, (0,0) is an evolutionarily stable point, (0,1) and (1,1) are saddle points, and (1,0) is an unstable point. This indicates that although universities may theoretically benefit from cooperation, the system ultimately stabilizes at a state where both parties do not cooperate due to the lack of incentive for the social group to open up.
(3) When the total payoff of cooperation among universities is negative, while the baseline net payoff of openness for social groups is negative but the total payoff is positive, i.e., $L_{b}-C_{A}^{S}+W+P_{c}+S\zeta +R^{e}\xi <\text{0}$, $D-C_{\text{0}}^{C}+F_{\text{1}}+F_{\text{2}}+M+P_{s}+S\lambda <\text{0}$, $D-C_{\text{0}}^{C}+F_{\text{1}}+F_{\text{2}}+M+P_{s}+S\lambda +R^{e}(\text{1}-\xi )>\text{0}$, as shown in Fig 5. 1(c). At this point, (0,0) is an evolutionarily stable point, (0,1) is an unstable point, and (1,0) and (1,1) are saddle points. This indicates that despite the social group’s willingness to open, the system still tends toward a non-cooperative state due to insufficient motivation for university cooperation.
(4) When the total benefit of university cooperation is positive while the total benefit of social group openness is negative, i.e., $L_{b}-C_{A}^{S}+W+P_{c}+S\zeta +R^{e}\xi >\text{0},D-C_{O}^{C}+F_{\text{1}}+F_{\text{2}}+M+P_{s}+S\lambda +R^{e}(\text{1}-\xi )<\text{0}$, as shown in Fig 5. 1(d),(0,0) is an evolutionarily stable point, (0,1) and (1,0) are saddle points, and (1,1) is an unstable point. This indicates that although universities have a willingness to cooperate, constrained by the conservative attitudes of social groups, the system cannot achieve collaborative cooperation.

#### 3.2.4. The influence of parameter changes on the stable equilibrium result of system evolution in the first case.

As mentioned above, when the cooperative benefit parameters between universities and social groups satisfy:


$$L_{b}-C_{A}^{S}+W+P_{c}+S\zeta <\text{0},\;L_{b}-C_{A}^{S}+W+P_{c}+S\zeta +R^{e}\xi >\text{0}\;$$

$$D-C_{O}^{C}+F_{\text{1}}+F_{\text{2}}+M+P_{s}+S\lambda <\text{0},\;$$

$$D-C_{O}^{C}+F_{\text{1}}+F_{\text{2}}+M+P_{s}+S\lambda +R^{e}(\text{1}-\xi )>\text{0}$$

There are two stable strategies in the evolutionary game between the two sides: (maintenance, conservative) and (cooperation, open).Which state the system ultimately converges to is determined by the magnitudes of the area $S_{\text{1}}$ of quadrilateral OUVW and the area $S_{\text{2}}$ of quadrilateral OVWX:When $S_{\text{1}}<S_{\text{2}}$, the probability of the system converging to the equilibrium point (0,0) is greater than the probability of converging to the equilibrium point (1,1); When $S_{\text{1}}>S_{\text{2}}$, the probability of the system converging to the equilibrium point (1,1) is greater than the probability of converging to the equilibrium point (0,0); When $S_{\text{1}}=S_{\text{2}}$ the probabilities of the system converging to two equilibrium points are equal.By analyzing the factors affecting the area of quadrilateral OUVW or OVWX, it can be converted into analyzing the influencing factors of the system’s evolutionary stable strategy under the first scenario. The calculation yields:


$$\begin{array}{c} S_{\text{1}}=\frac{\text{1}}{\text{2}}[\frac{C_{A}^{S}-L_{b}-W-P_{c}-S\zeta }{R^{e}\xi }+\frac{D-C_{O}^{C}+F_{\text{1}}+F_{\text{2}}+M+P_{s}+S\lambda }{R^{e}(\text{1}-\xi )}] \end{array}$$

Based on the parameters affecting the area of quadrilateral OUVW, the following proposition is derived through analysis:

Proposition 1: When the net benefit benchmark value of university cooperation is smaller (that is, the more $L_{b}-C_{A}^{S}+W+P_{c}+S\zeta$ it is negative), and the net benefit benchmark value of social group openness is larger (that is, the closer $D-C_{O}^{C}+F_{\text{1}}+F_{\text{2}}+M+P_{s}+S\lambda$ it is positive), the probability that the system converges to (maintains, conserves) is greater. Conversely, when the net benefit benchmark value of university cooperation is larger and the net benefit benchmark value of social group openness is smaller, the probability of the system converging to (cooperation and openness) is greater.

Proof: When other factors are constant, partial derivatives will be taken with respect to the relevant parameters respectively. Conclusion by conditions: $C_{A}^{S}-L_{b}-W-P_{c}-S\zeta >\text{0},\;D-C_{O}^{C}+F_{\text{1}}+F_{\text{2}}+M+P_{s}+S\lambda <\text{0}$

so:


$$\frac{\partial S_{\text{1}}}{\partial (C_{A}^{S})}=\frac{\text{1}}{\text{2}R^{\epsilon }\xi }>\text{0}$$

$$\frac{\partial S_{\text{1}}}{\partial (L_{b})}=-\frac{\text{1}}{\text{2}R^{e}\xi }<\text{0}$$

$$\frac{\partial S_{\text{1}}}{\partial (C_{O}^{C})}=-\frac{\text{1}}{\text{2}R^{e}(\text{1}-\xi )}<\text{0}$$

$$\frac{\partial S_{\text{1}}}{\partial (D)}=\frac{\text{1}}{\text{2}R^{e}(\text{1}-\xi )}>\text{0}$$

It can be seen from this that an increase in the cooperation cost of colleges and universities $C_{A}^{S}$ will increase $S_{\text{1}}$, thereby increasing the probability that the system converges to (maintain, conservative). An increase in the loss of reputation of colleges and universities $L_{b}$ will reduce $S_{\text{1}}$, thereby increasing the probability that the system converges to (cooperation, openness); An increase in the open cost of social groups $C_{O}^{C}$ will reduce $S_{\text{1}}$, thereby increasing the probability that the system converges to (cooperation, openness); An increase in the direct benefits of social groups D will increase $S_{\text{1}}$ thereby increasing the probability that the system converges to (maintain, conservative).

This analysis result indicates that in a bistable state, the government can influence the probability of the system converging to the ideal state (cooperative, open) by adjusting relevant parameters. Specifically, measures such as reducing the cooperation costs among universities, increasing the damage to their reputation, lowering the opening costs of social groups, and controlling the direct benefits of social groups all contribute to increasing the probability of the system converging to an ideal collaborative state.

Proposition 2: When the benchmark benefit of universities choosing the “maintenance” strategy is smaller, while the benchmark benefit of social groups choosing the “open” strategy is larger, the probability of the system converging to (maintenance, conservative) is greater. Conversely, the greater the probability that the system converges to (cooperation and openness).

Proof: Partial derivatives of $S_{\text{1}}$ with respect to $L_{b}$ and D respectively, from the conditions, it can be known that:


$$\begin{array}{c} \frac{\partial S_{\text{1}}}{\partial L_{b}}=-\frac{\text{1}}{\text{2}R^{e}\xi }<\text{0},\;\frac{\partial S_{\text{1}}}{\partial D}=\frac{\text{1}}{\text{2}R^{e}(\text{1}-\xi )}>\text{0} \end{array}$$

It can be seen that $S_{\text{1}}$ is a decreasing function of $L_{b}$ and an increasing function of D. When other factors remain unchanged, the smaller the benchmark benefit $L_{b}$ of universities choosing the “maintenance” strategy and the larger the benchmark benefit D of social groups choosing the “open” strategy, the greater the probability that the system converges to the equilibrium point (0,0), that is, neither side cooperates. Conversely, the greater the probability that the system converges to (1,1).

Proposition 3: When the “free-rider” benefits of universities are greater and those of social groups are smaller, the probability that the system converges to (maintain, conservative) is also greater. Conversely, the greater the probability that the system converges to (cooperation and openness).

Proof: Take the partial derivatives of $S_{\text{1}}$ with respect to E and $G_{\text{2}}$ respectively. From the conditions, it can be known that:


$$\frac{\partial S_{\text{1}}}{\partial E}=\frac{C_{A}^{S}-L_{b}-W-P_{c}-S\zeta }{\text{2}R^{e\text{2}}\xi^{\text{2}}}>\text{0},\;\frac{\partial S_{\text{1}}}{\partial G_{\text{2}}}=-\frac{D-C_{O}^{C}+F_{\text{1}}+F_{\text{2}}+M+P_{s}+S\lambda }{\text{2}R^{e\text{2}}(\text{1}-\xi {)}^{\text{2}}}<\text{0}$$

It can be seen that $S_{\text{1}}$ is an increasing function of E and a decreasing function of $G_{\text{2}}$. When other factors remain unchanged, the greater the university’s “free-riding” benefit E and the smaller the social group’s “free-riding” benefit $G_{\text{2}}$, the higher the probability that the system converges to the equilibrium point (0,0); conversely, the higher the probability that the system converges to (1,1).

Proposition 4: When the cost of cooperation among universities is greater and the cost of openness among social groups is smaller, the probability of the system converging to (maintaining, conservative) is also greater. Conversely, the greater the probability that the system converges to (cooperation and openness).

Proof: Partial derivatives of $S_{\text{1}}$ with respect to $C_{A}^{S},C_{O}^{C}$respectively, from the conditions, it can be known that:


$$\frac{\partial S_{\text{1}}}{\partial C_{A}^{S}}=\frac{\text{1}}{\text{2}R^{e}\xi }>\text{0},\;\frac{\partial S_{\text{1}}}{\partial C_{O}^{C}}=-\frac{\text{1}}{\text{2}R^{e}(\text{1}-\xi )}<\text{0}$$

It can be seen that $S_{\text{1}}$ is an increasing function of $C_{A}^{S}$ and a decreasing function of $C_{O}^{C}$. When other factors remain unchanged, the higher the cost of university cooperation $C_{A}^{S}$ and the lower the cost of social group openness $C_{O}^{C}$, the greater the probability that the system converges to the equilibrium point (0,0). Conversely, the greater the probability that the system converges to (1,1).

Proposition 5: The Impact of Government Reward and Punishment Intensity on the System’s Convergence Probability

Proof: Taking the partial derivatives of $S_{\text{1}}$ with respect to the government reward and punishment parameters respectively:


$$\begin{array}{c} \frac{\partial S_{\text{1}}}{\partial P_{c}}=-\frac{\text{1}}{\text{2}R^{e}\xi }<\text{0},\;\frac{\partial S_{\text{1}}}{\partial P_{s}}=\frac{\text{1}}{\text{2}R^{e}(\text{1}-\xi )}>\text{0}\\ \frac{\partial S_{\text{1}}}{\partial W}=-\frac{\text{1}}{\text{2}R^{e}\xi }<\text{0},\;\frac{\partial S_{\text{1}}}{\partial \left(F_{\text{1}}+F_{\text{2}}\right)}=\frac{\text{1}}{\text{2}R^{e}(\text{1}-\xi )}>\text{0} \end{array}$$

It can be seen that increasing the reward $P_{c}$ and punishment W for universities will reduce $S_{\text{1}}$, thereby increasing the probability that the system converges to (1,1); while increasing the reward $P_{c}$ and punishment $F_{\text{1}}+F_{\text{2}}$ for social groups will increase $S_{\text{1}}$, which in turn raises the probability that the system converges to (0,0). This result indicates that under the bistable state, the government’s reward and punishment policies need to adopt differentiated strategies for different game participants.

### 3.3. Numerical simulation

This paper performs numerical simulations to examine scenarios where universities and society initially exhibit a high willingness to cooperate (x = 0.9, y = 0.9). By adjusting the government grant value S within the system, we observe changes in the sensitivity of the system’s evolutionary path.

**Fig 6:**
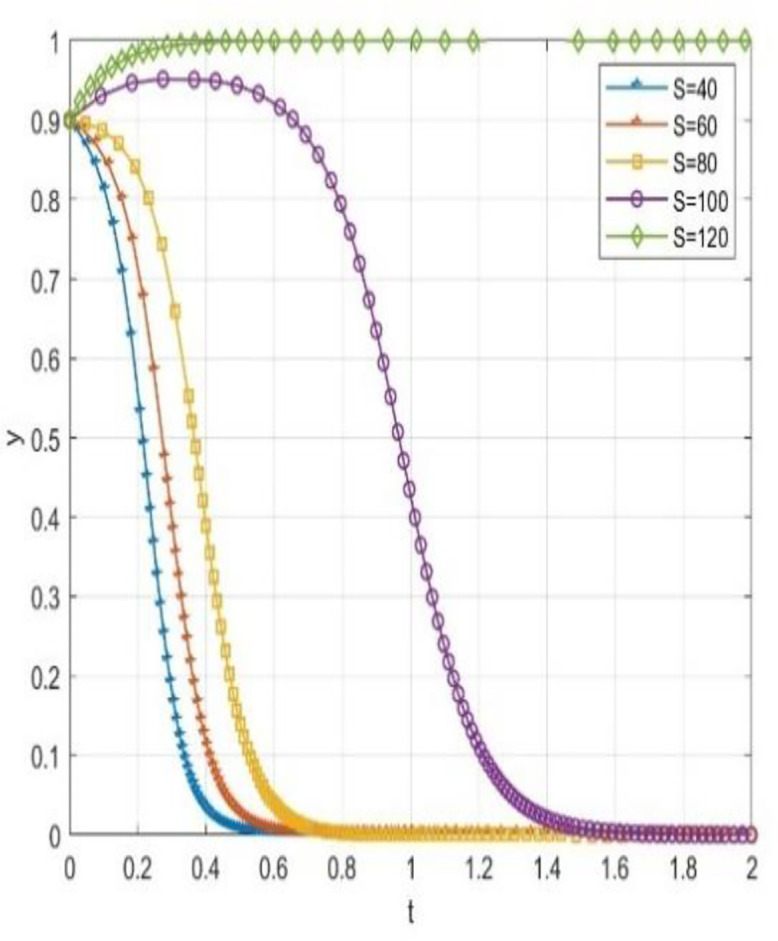
The impact of government funding on the probability of social openness.

The simulation results indicate that even when universities and society exhibit a strong willingness to cooperate, variations in government subsidies significantly influence the system’s evolution. Fig 5 illustrates the trajectory of the probability (x) of universities adopting cooperative strategies at different subsidy levels. Concurrently, Fig 6 displays the evolution path of the probability (y) of social institutions adopting open strategies. When the government grant S is high (S = 120), the system converges strongly. The strategic probabilities for both universities and social organizations quickly and steadily approach the (1,1) equilibrium point from an initially high level without any decline. This suggests that high subsidies adequately cover collaborative costs and yield substantial net benefits, making the “cooperative-open” strategy strictly dominant and maintaining system stability in an ideal collaborative state. As subsidy intensity decreases, the system’s evolution trajectory diverges significantly, and coordination probability drops markedly with reduced subsidies.

Notable even if there is good cooperation, reduction in subsidy level may alter the direction of evolution and cause the system to deviate from the ideal collaborative state into a non-cooperative state. This phenomenon shows that the static reward mechanism has a certain degree of limitation: The school-community collaboration system is easily affected by the decline in government subsidies, and the relationship of cooperation is closely related to the continuous and stable input of external resources. This obvious “subsidy dependence” shows that even under static conditions, the participants, though there is a good cooperative basis, is lacking in endogenous stability, the system of evolution is highly sensitive to changes in the government of the resource input.

This analysis indicates that static reward methods cannot make the intrinsic stability of collaborative systems fundamentally. The effectiveness of these systems is clearly policy-based, and their lasting effectiveness depends on the continuous and high-intensity supply of external resources. From a view of dynamic development, the incentive mechanisms are to be reformed immediately and a dynamic linked mechanism, closely correlated with the performance, should be generated.

In talent development practices, the reward mechanism involves providing non-material incentives to colleges and universities by granting one-time honorary titles such as “Demonstration Unit for Mental Health Education” and “Outstanding Team for Psychological Crisis Intervention.” The punishment mechanism includes measures such as public criticism, point deductions in assessments for colleges and universities, and economic penalties, suspension, or revocation of campus mental health service qualifications for social institutions. While the “static reward and static punishment” mechanism may be feasible during the collaborative start-up phase, its inherent lack of incentives, flexibility, and innovation, coupled with the significant one-off nature of its measures, severely limits the high-quality development of the school-community collaborative psychological education system in terms of professionalization, systematization, and sustainability.

## 4. Analysis of the evolution game model of dynamic reward and punishment mechanism under government intervention

The government-involved school-client collaborative education system for college students’ mental health education, although the static reward mechanism can stimulate cooperation to a certain extent, it lacks flexibility and adaptability and is difficult to cope with complex and changing real-life environments.In order to fundamentally solve the coordination dilemma of “the government will move if it promotes, and if the government does not push, it will stop”, this chapter constructs a “reputation incentives”, this chapter builds a dynamic reward and punishment system with the “reputation incentives-performance appropriation” linkage system as the core. This system realizes progressive dynamic update of reputation by quantifying collaborative behaviors into accumulable reputation incentives and constructing multi-level reputation levels. This design meets the core demands of dynamic capabilities——Teece (2007) confirmed that dynamic incentives need to focus on capability improvement rather than pure resource investment. Linking reputation incentives with resource integration, service reconstruction and other capability indicators can significantly improve collaboration stability. On this basis, ζ(x) and λ(y) make adjustments, making it a degree of university cooperation (x)and social openness(y)function, establishing a dynamic relationship between performance and return [38].

This chapter constructs a game model of three situations: dynamic reward-static punishment, static reward-dynamic punishment and completely dynamic. Through system stability analysis and numerical simulation, the effectiveness of this dynamic linkage system in promoting the evolution of the system to an efficient and stable mental health service community is tested.

### 4.1. Dynamic reward and static punishment situation model

This section builds a “dynamic reward-static punishment” hybrid mechanism model. Among them, the intensity of government rewards to universities and society is dynamically related to their degree of cooperation and openness in collaborative work such as co-construction of mental health courses and psychological crisis intervention, forming a positive feedback loop.

The intensity of government incentives to universities and society is related to the degree of cooperation and openness in collaborative education work such as co-construction of mental health courses, psychological crisis prevention and intervention, and sharing of psychological counseling services. Specifically, the government’s dynamic reward function for colleges and universities is set as $R\left(x\right)=\left(\text{1}-x\right)\zeta S+xP_{c}$. The dynamic reward function for society is set to $R\left(y\right)=\left(\text{1}-y\right)\lambda S+yP_{s}$. This design not only responds to the core demands of universities and social institutions: universities obtain sufficient material resources in the early stages of cooperation **(**(1−x)ζS**)** to start the project, and after the cooperation deepens, more reliance will be placed on reputation incentives (the differentiated needs of **(**$xP_{c}$**)**, which also provides social institutions with a smooth path from obtaining commercial returns to improving brand reputation [39].

At the same time, the penalty mechanism remains statically set, and fixed constraints W are implemented for universities that adopt maintenance strategies and fixed constraints $F_{\text{1}}$ and $F_{\text{2}}$ are implemented for social groups that adopt conservative strategies. Then the expected benefits and replication dynamic equations of universities and society are:

The expected benefits of universities that choose to cooperate are:


$$\pi_{x}=(y-\text{1})(C_{A}^{S}+G_{\text{2}}-p_{c}x+S\zeta (x-\text{1}))+y(E-C_{A}^{S}+R^{e}\xi +p_{c}x-S\zeta (x-\text{1}))$$

The expected benefits of universities that choose not to cooperate are:


$$\pi_{\text{1}-x}=(y-\text{1})(G_{\text{2}}+L_{b}+W)-y(L_{b}-E+W)$$

The dynamic equation for university replication is as follows:


$$\frac{dx}{dt}=-x(x-\text{1})(L_{b}-C_{A}^{S}+W+S\zeta +p_{c}x+R^{e}\xi y-Sx\zeta )$$

The expected benefits of the social group that chooses to cooperate are:


$$\pi_{y}=(x-\text{1})(C_{O}^{C}-D-P_{s}y+S\lambda (y-\text{1}))-x(C_{O}^{C}-D-P_{s}y+R^{e}(\xi -\text{1})+S\lambda (y-\text{1}))$$

The expected benefit of a social group that chooses not to cooperate is:


$$\pi_{\text{1}-y}=(x-\text{1})(F_{\text{1}}+F_{\text{2}}+M)-x(F_{\text{1}}+F_{\text{2}}+M)$$

The social replication dynamic equation is as follows:


$$\frac{dy}{dt}=-y(y-\text{1})(D-C_{O}^{C}+F_{\text{1}}+F_{\text{2}}+M+S\lambda +R^{e}x+P_{s}y-S\lambda y-R^{e}x\xi )$$

It can be concluded that there are 5 replication dynamic equilibrium points in system evolution: (0,0), (0,1), (1,0), (1,1), $\left(x^{*},y^{*}\right)$.and $\left(x^{*},y^{*}\right)=(x^{*}=\frac{D-C_{O}^{C}+F_{\text{1}}+F_{\text{2}}+M+P_{s}y-S\lambda (y-\text{1})}{R^{*}(\xi -\text{1})},y^{*}=\frac{C_{A}^{S}-L_{b}-p_{c}x+W(x-\text{1})+S\zeta (x-\text{1})}{R^{e}\xi })$

at X*∈[0,1],Y*∈[0,1] time, according to the Jacobian matrix local stability analysis method, the stability analysis of the five equilibrium points of the system is carried out, and the results are shown in the Table 8.

**Table 8:** Local Stability of the Equilibrium Point.

equilibrium point	Tr(J)symbol	Det(J)symbol	result
(0,0)	+	–	ESS
(0,1)	+	+	unstable
(1,0)	+	+	unstable
(1,1)	+	–	ESS

**Fig 7:**
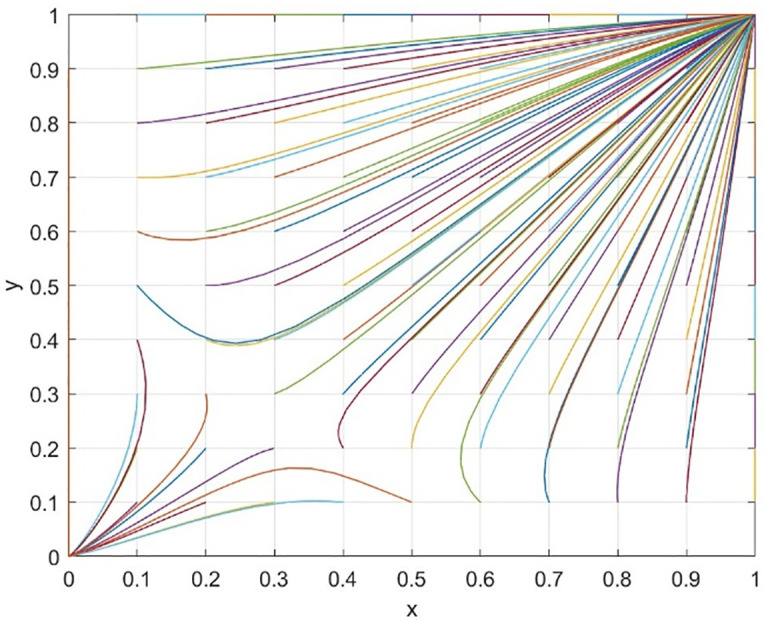
Evolutionary processes under dynamic reward and static punishment mechanisms.

Simulation is used below to prove that the above results and parameter settings remain unchanged. Fig 7 shows the evolution process under the dynamic reward-static punishment mechanism, presenting a trend where the probability of university cooperation (x) and the probability of social openness (y) converge towards a stable equilibrium state.

#### 4.1.1 The influence of the intensity of the Two reward models on the evolution path.

**Fig 8:**
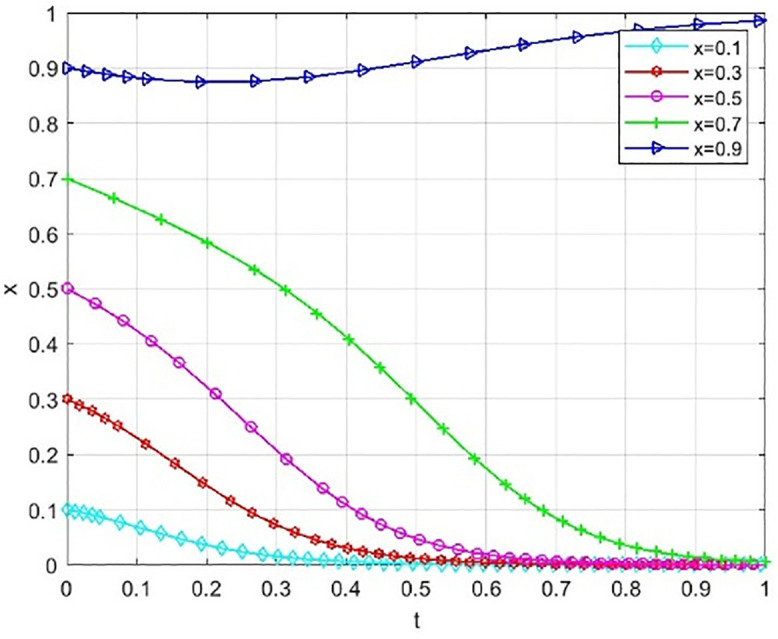
The impact of initial conditions on evolutionary outcomes.

(1) S and pc、ps the impact on the strategies of both sides under different initial intentions (Fig 8).

**Fig 9:**
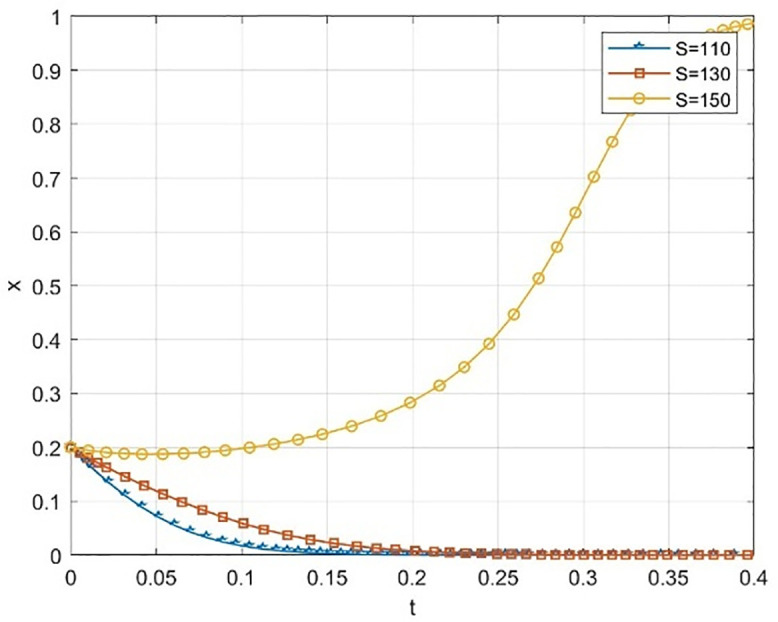
The impact of government funding on universities’ probability of taking the initiative when cooperation willingness is low.

**Fig 10:**
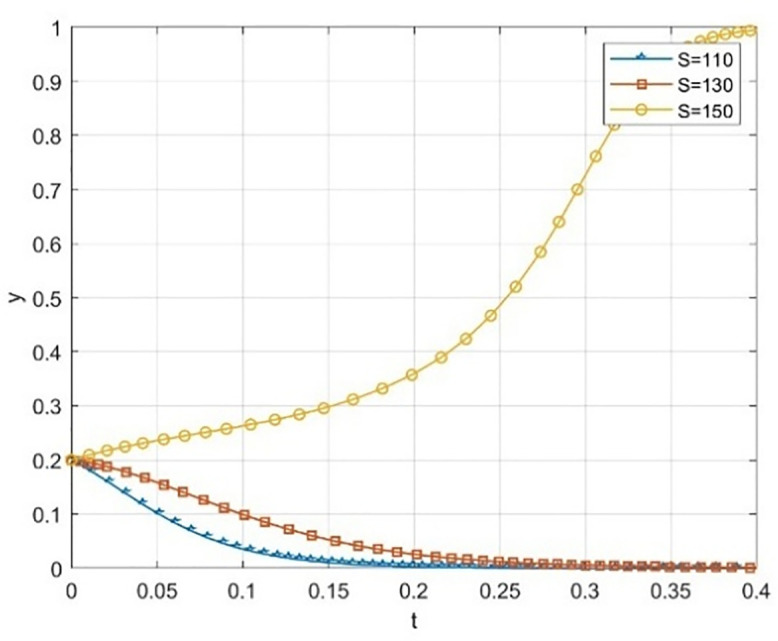
The impact of low cooperation willingness on the probability of government funding being open to society.

**Fig 11:**
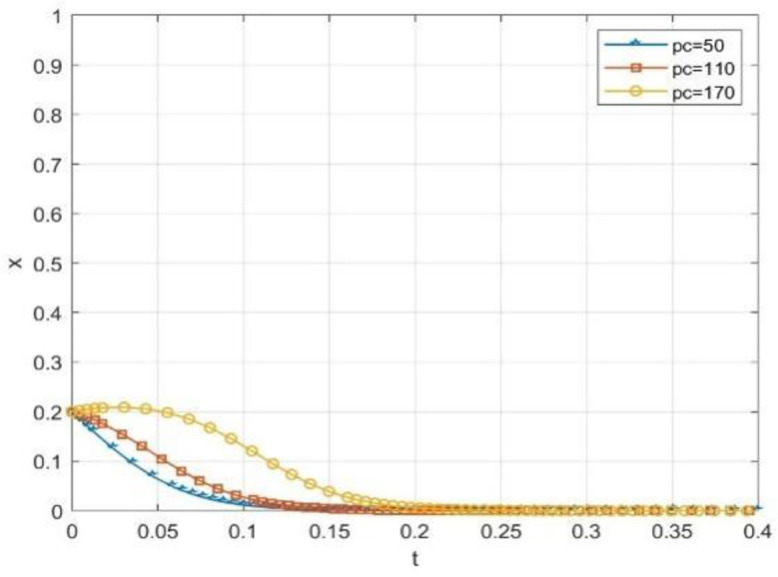
The effect of reputation subsidies on the probability of universities taking the initiative when cooperation willingness is low.

**Fig 12:**
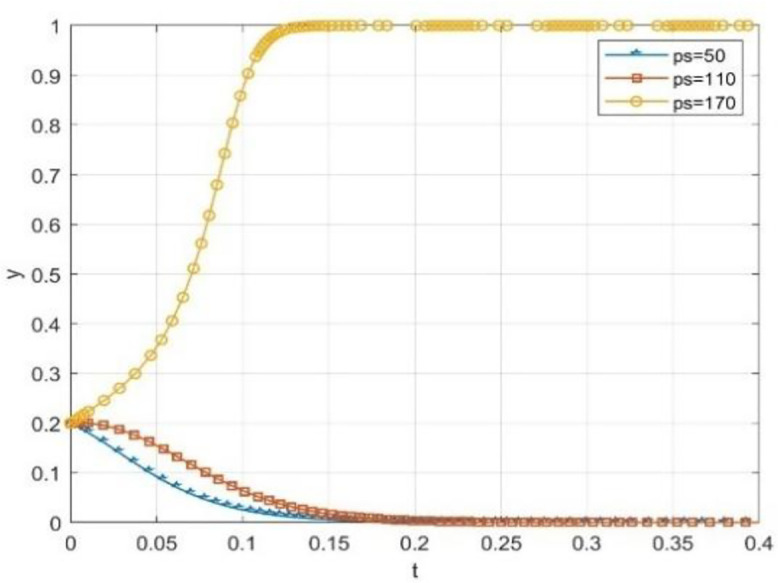
The effect of reputation subsidies on the probability of social openness when cooperation willingness is low.

Numerical simulation is conducted in a difficult situation where both universities and society have a relatively low initial willingness (x = 0.2, y = 0.2) to cooperate.We investigated the different evolutionary paths of the system as it moved toward a collaborative state, focusing on the reputation incentives Ps of social organizations and Pc of universities. The findings are presented in Figs 9–12.

The simulation results in Figs 9 and 10 indicate that within the dynamic reward-static punishment framework, increasing government subsidy S significantly enhances the system’s evolution prospects, especially when both the school’s and society’s initial cooperation willingness is low (x = 0.2, y = 0.2). As S rises, the cooperation probability (x) of universities and the openness probability (y) of society exhibit a marked upward acceleration. At S = 150, the system swiftly transitions from a low-level “maintenance-conservative” state to a high-level collaborative stable state. This finding underscores the essential role of material resource input in overcoming initial deadlocks and motivating participation during the collaborative start-up phase.

Figs 11 and 12 respectively illustrate the impact of increasing the reputation incentive Pc for universities and the reputation incentive Ps for social organizations, each under the same low initial intention. The simulation results reveal that although boosting the intensity of reputation incentives positively influences cooperation, both the incentive effect and the evolution speed are notably weaker than those achieved through a smaller increase in government subsidies S. This suggests that in the early stages, when cooperation is not well-established, participants are more responsive to immediate, tangible material incentives, while the effectiveness of long-term reputation capital accumulation experiences a delay. Moreover, when Pc and Ps are both raised to the same level, reaching Pc = Ps = 170, the cooperation probability for universities fluctuates before settling at a low level, while the openness probability of society remains at a high level. This is far from society’s harsh treatment of reputation is much more severe than universities.

Comprehensive compared with the simulation results of Quantumolc. S: Under a dynamic reward-static punishment mechanism, the government intervention strategy should prioritize financial subsidies to enhance the initial willingness to cooperate. The material resources should be able to play the role of the “initial lever” role to promote the emergence of collaborative behavior and to lay the foundation and guarantee the progress of systems reaching the ideal state. At this time, reputation incentive is more supportive. This finding provides a reference basis for the government to make decisions and allocate incentive tools at different stages of collaboration. Material incentives should be highlighted above all else at present. As cooperation deepens, more attention should be paid to non-material incentives such as reputation to ensure efficient allocation of resources and continuous progress of the collaborative system.

**Fig 13:**
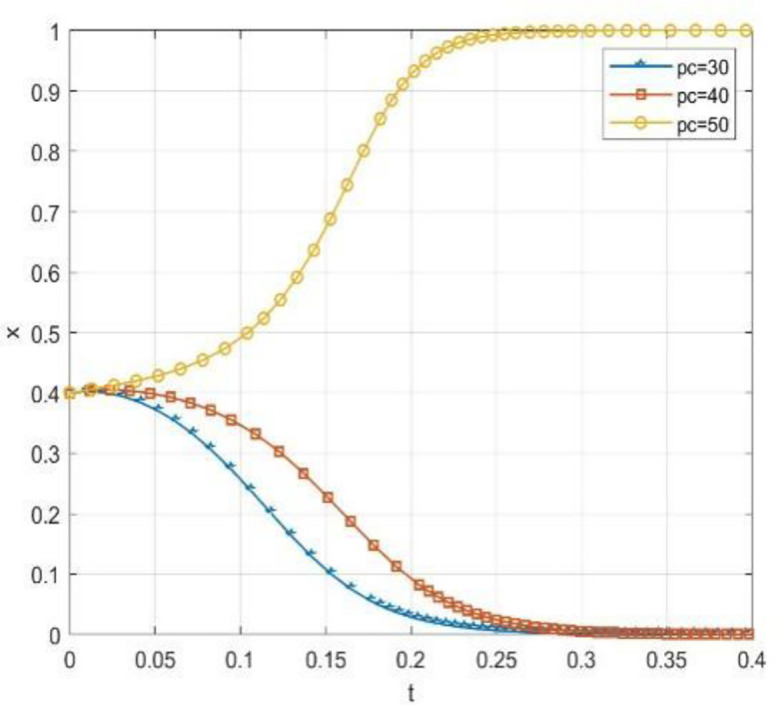
The effect of reputation rewards on the probability of universities taking the initiative under moderate cooperation willingness.

**Fig 14:**
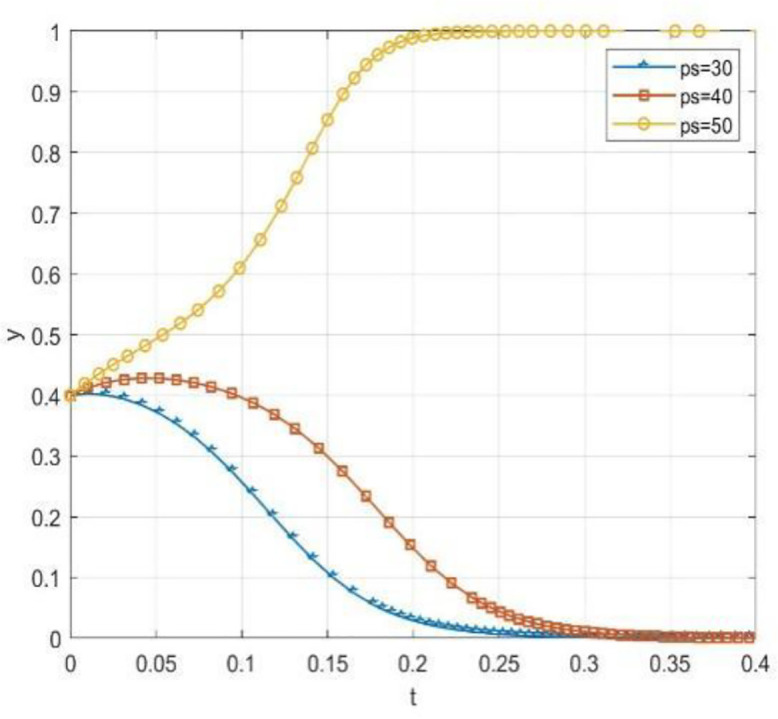
The effect of reputation rewards on the probability of social openness under moderate cooperation willingness.

The effectiveness of incentive tools is generally changing with the system’s collaborative state. To explore this boundary condition, this paper simulates the evolution path of the school and society’s initial willingness to cooperate at the higher level of x = 0.4 and y = 0.4. It special care the role of reputation incentives moderating effect. The results are shown in Figs 13, 14.

The simulation results show that when the cooperation relationship between universities and social organizations is not very reluctant, reputation incentives can effectively maintain and promote such cooperation. As shown in the figure, even if the government-provided reputation incentive Pc is only 50, the willingness of universities to cooperate is still quite high, and the mutual willingness to cooperate is also relatively high. When Ps is equal to 50, it is also the case that there is a willingness to engage in social cooperation. Different from the important “initiating” role of the government subsidy (S) in the scenario of low willingness to act, at this stage, a slight increase in the reputation incentive can bring substantial marginal benefit. This can consolidate and improve the synergy level effectively without relying on large-scale continuous financial subsidies. In the implementation process of “Comprehensive Nurturing Model” service mode in Yangcheng County, Shanxi Province, China under the construction of the pilot project of supporting children in difficult circumstances by the state, it provides practice evidence. This model tells that incentive tools is have differentiated adaptedes at different cooperations level.

**Fig 15:**
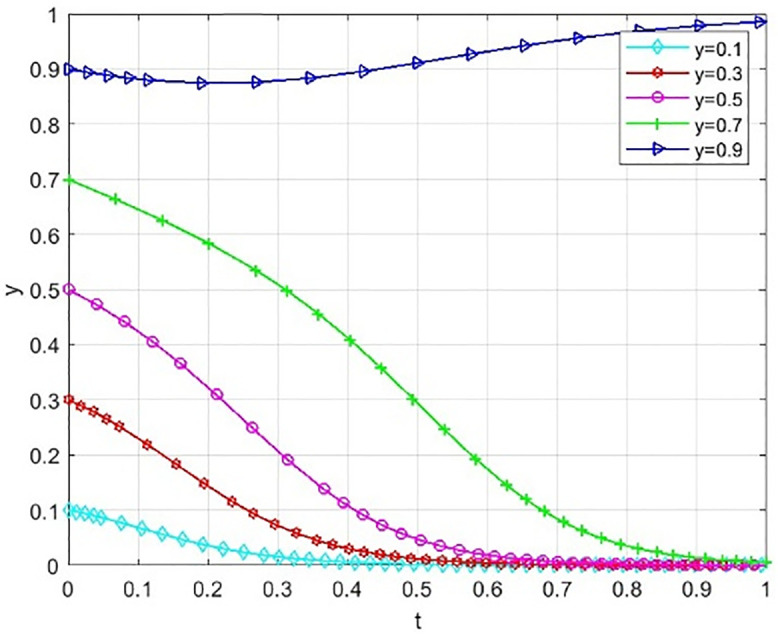
The impact of initial conditions in higher education institutions on evolutionary outcomes.

From Fig 13–15 shows the simulation results indicate a key finding that the “dynamic reward static punishment” mechanism highlights the complementary effects and stage specific advantages of government subsidies and reputation incentives. The period of the collaborative system, or if one or both parties are not willing to cooperate, at this stage, using government subsidies can break the deadlock and stimulate the other party to participate in the cooperation. As cooperation deepens, and participation will increase, it will become the most important way to achieve a goal, the way to gain the long-term reputation capital, so as to maintain deep collaboration and system stability. This finding confirms the logic of the Design of the dynamic mechanism, which is to adaptively transform the incentive methods to realize the evolution from “resource-driven” to “endogenous identification”, and thus more effectively solve the collaborative dilemma of “action only when the government promotes and stagnation when it does not”.

**Fig 16:**
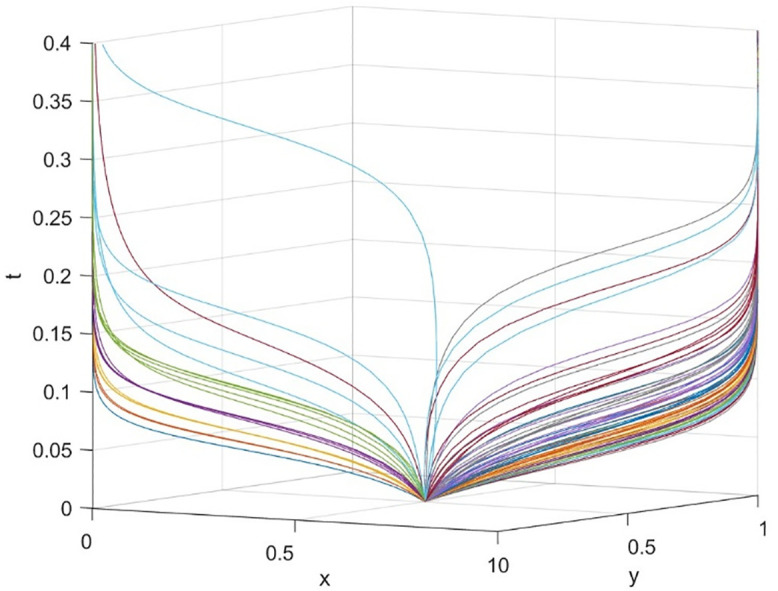
Evolutionary processes under different dynamic reward coefficient combinations.

(2) The influence of different proportions of reward intensity coefficients on the strategies of both sides (Fig 16).

This paper delves into the dynamic reward mechanism by examining how various combinations of the dynamic distribution coefficients for government subsidies—namely, the dynamic reward intensity coefficient λ for social organizations and the dynamic reward intensity coefficient ζ for universities—affect the system’s evolution efficiency. Fig 13 provides a macro view of the system’s convergence speed across different resource configurations by analyzing 81 combinations of λ and ζ, ranging from 0.1 to 0.9, with color gradients representing their sum. The findings reveal that even when the total incentive resources (λ + ζ) are similar, the system’s evolution speed towards an efficient and collaborative state varies. This suggests that the allocation structure of incentive resources between universities and social organizations is a crucial policy variable.

**Fig 17:**
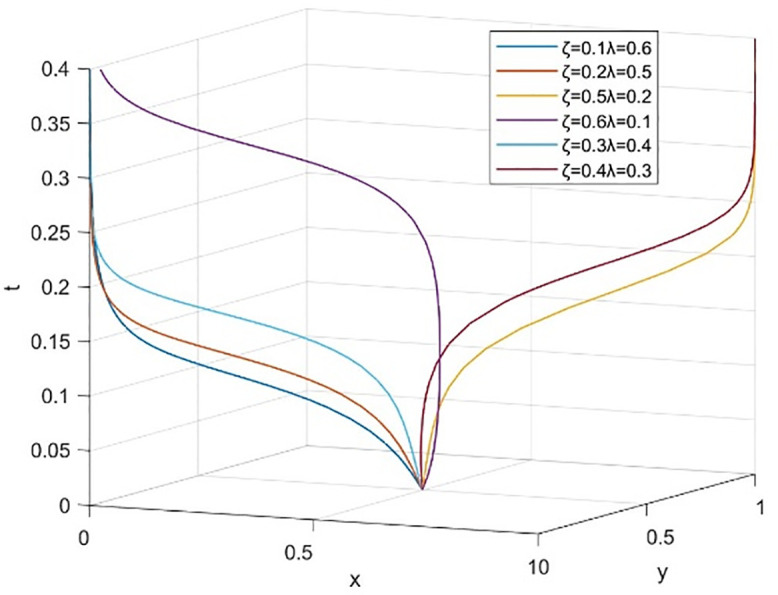
Effect of different reward coefficient combinations on evolutionary outcomes when λ + ζ = 0.7.

**Fig 18:**
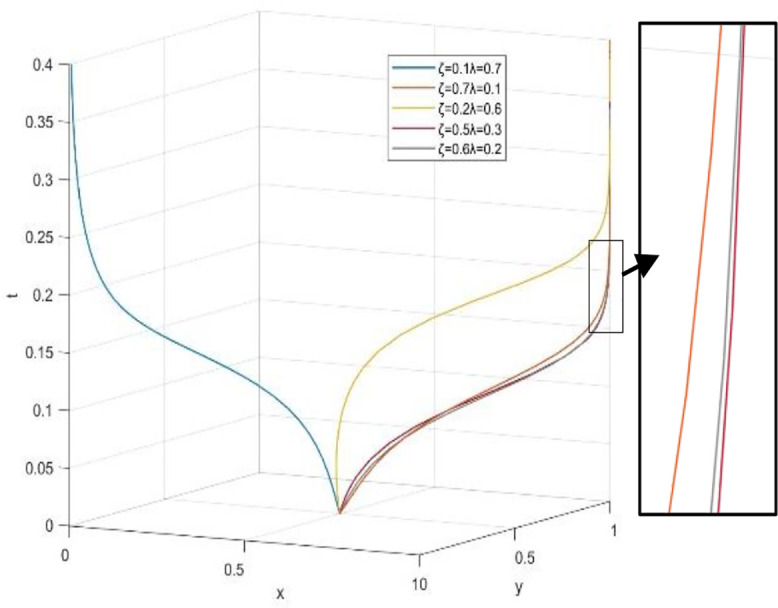
Effect of different reward coefficient combinations on evolutionary outcomes when λ + ζ = 0.8.

The research aims to identify efficient incentive resource allocation schemes by examining combinations with a reduced total amount of incentive resources (λ + ζ). Fig 17 illustrates a series of state evolution paths for λ + ζ = 0.7, while Fig 18 depicts these paths for λ + ζ = 0.8.

The simulation results reveal an important asymmetric incentive revelation: the process of dynamic subsidies benefiting universities clearly works better than universities not benefitting for the evolution of driving systems. When configured at (λ = 0.2, ζ = 0.5), the system evolves most rapidly. Even in the case of heavy policy support for universities (λ = 0.1, ζ = 0.7), the system is still able to reach the ideal state. This is consistent with the research conclusion on the audit ecosystem: Concentrate resources to support the main body (universities and high-quality audit parties) to avoid the dispersion of resources caused by “adversarial incentives” and more efficiently cultivate the self-sustaining ability of the collaborative ecosystem [40]. When subsidies mainly benefit society (for example, λ = 0.7, ζ = 0.1), even if the total amount of funds is increased, the system’s evolution speed is slow or even fails. This highlights the importance of providing sufficient incentives in terms of resources for universities at the initial stage of cooperation to start the cooperation smoothly. Under the condition of limited resources, dynamic subsidies are more likely to favor universities (higher ζ) because they can better drive the entire collaborative system compared to an average distribution or a focus on society. This phenomenon is probably due to universities playing a leading role in the collaborative education network and having more specialized investment costs. Univarsities will be able to get more significant marginal incentives.

### 4.2. Static reward and dynamic punishment scenario model

This section constructs a hybrid mechanism model of “static reward - dynamic punishment”. The intensity of government punishment towards universities and society is dynamically related to the degree of openness and cooperation in mental health education. When colleges and universities actively fulfill their primary responsibility for mental health education (such as systematically offering psychological courses and standardizing the operation of psychological counseling centers), or when social institutions voluntarily open their psychological counseling platforms and share psychological assessment tools, the penalties imposed by the government will be correspondingly reduced. Conversely, when the participation of the participants in the psychological education collaborative network is low and resources are closed, the intensity of punishment gradually increases. This design embodies the principle of matching punishment with the degree of coordination.

Let the dynamic penalty function of the government for universities be W(x)=(1-x)W, and the dynamic penalty function for society be $p_{c}$. This progressive punishment design not only provides sufficient autonomy space for high-reputation and deeply involved subjects, but also effectively maintains the stability of the educational community through a gradually upgraded constraint mechanism for low-participation subjects, achieving an organic balance between institutional autonomy and systematic accountability. Meanwhile, the reward mechanism remains statically set. The government’s reputation incentives $p_{c}$ for universities, reputation incentives $P_{s}$ for social groups, and financial allocations S all adopt fixed parameters to provide stable positive expectations for collaborative behavior. Then, the dynamic equations of expected returns and replication for universities and society are:The expected returns of the universities that choose to cooperate are:


$$\pi_{x}=y\left(\xi R^{e}-C_{A}^{S}+E+\zeta S+p_{c}\right)+(\text{1}-y)\left(-G_{\text{2}}-C_{A}^{S}+\zeta S+p_{c}\right)$$

The expected benefits of the universities that choose not to cooperate are:


$$\pi_{\text{1}-x}=y(E-L_{b}-(\text{1}-x)W)+(\text{1}-y)(-L_{b}-G_{\text{2}}-(\text{1}-x)W)$$

The dynamic equation for replication in colleges and universities is as follows:


$$\frac{dx}{dt}=-x(x-\text{1})(L_{b}-C_{A}^{S}+W+p_{c}+S\zeta -Wx+R^{e}\xi y)$$

The expected benefits of the social groups that choose to cooperate are:


$$\pi_{y}=x\left((\text{1}-\xi )R^{e}-C_{O}^{C}+D+\lambda S+P_{s}\right)+(\text{1}-x)\left(D-C_{O}^{C}+\lambda S+P_{s}\right)$$

The expected benefits for social groups that choose not to cooperate are:


$$\pi_{\text{1}-y}=x\left(-M-(\text{1}-y)F_{\text{1}}-(\text{1}-y)F_{\text{2}}\right)+(\text{1}-x)\left(-M-(\text{1}-y)F_{\text{1}}-(\text{1}-y)F_{\text{2}}\right)$$

The dynamic equation of social replication is as follows:


$$\frac{dy}{dt}=-y(y-\text{1})(D-C_{O}^{C}+F_{\text{1}}+F_{\text{2}}+M+P_{s}-F_{\text{1}}y-F_{\text{2}}y+S\lambda +R^{e}x-R^{e}x\xi )$$

It can be concluded that there are a total of five replication dynamic equilibrium points in the system evolution: (0,0), (0,1), (1,0), (1,1), $\left(x^{*},y^{*}\right)$. Among them $\left(x^{*},y^{*}\right)=(\frac{D-C_{O}^{C}+M+P_{s}+S\lambda -F_{\text{1}}(y-\text{1})-F_{\text{2}}(y-\text{1})}{R^{e}(\xi -\text{1})},-\frac{L_{b}-C_{A}^{S}+p_{c}+S\zeta -W(x-\text{1})}{R^{e}\xi })$

When X*∈[0,1] and Y*∈[0,1], the stability analysis of the five equilibrium points of the system was conducted based on the Jacobian matrix local stability analysis method. The results are shown in the Table 9.

**Table 9:** Local Stability of the Equilibrium Point.

Equilibrium Point	Sign of Tr(J)	Det(J) Sign	Result
(0,0)	+	–	ESS
(0,1)	N	–	Saddle Point
(1,0)	N	–	Saddle Point
(1,1)	+	–	ESS

**Fig 19:**
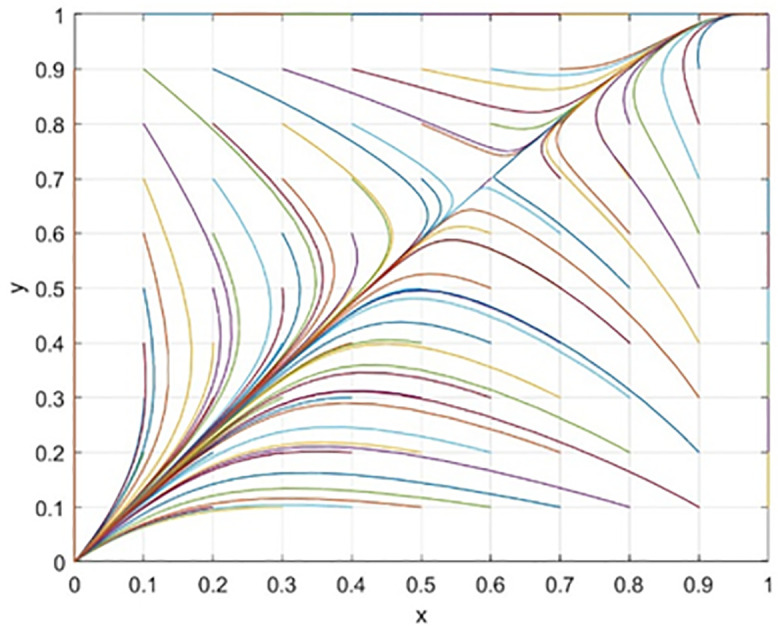
Evolutionary processes under static reward and dynamic punishment mechanisms.

**Fig 20:**
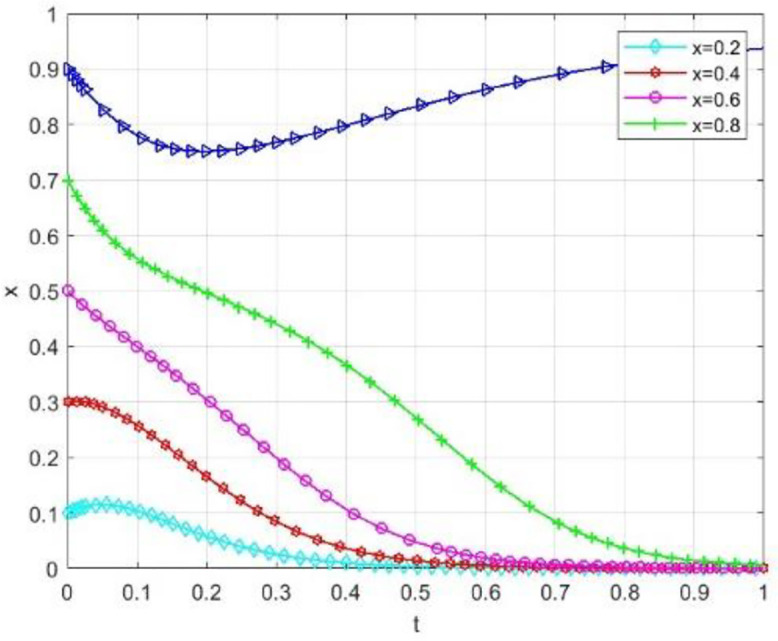
The impact of initial conditions in higher education institutions on evolutionary outcomes.

**Fig 21:**
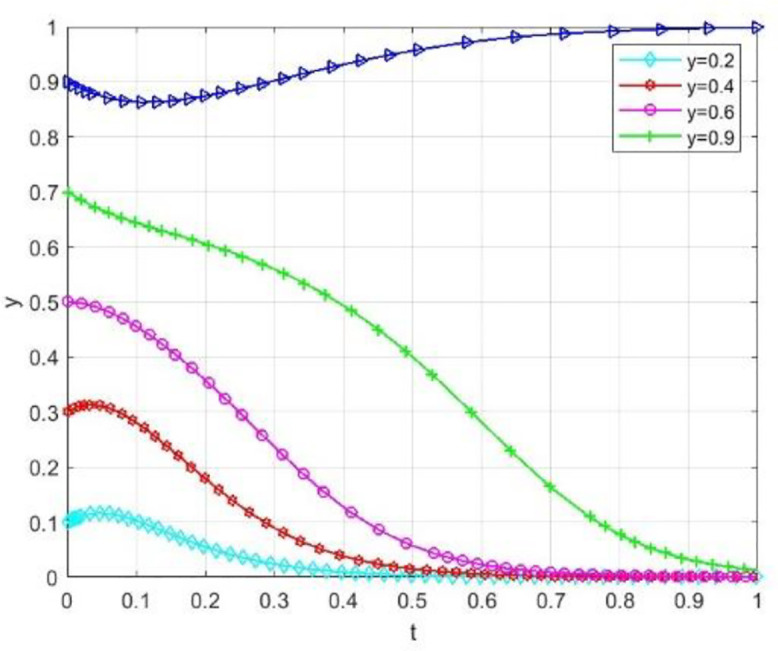
The impact of initial conditions on evolutionary outcomes.

The following simulation is used to prove that the parameter Settings of the above results remain unchanged (Figs 19–21).

### 4.3. Dynamic reward and dynamic punishment scenario model

This section constructs a fully dynamic two-way feedback system, where the intensity of government rewards and punishments for universities and society is dynamically correlated with the degree of openness and cooperation in mental health education. The government adopts the principle of differentiated incentives. When the willingness to cooperate is low, it enhances fiscal incentives through dynamic reward intensity coefficients ζ and λ. When the willingness to cooperate is high, the influence of the initial values of the main society on the evolutionary outcome gives reputation incentives to PC and PS, guiding the subjects to shift from “passive participation” to “active co-construction “ Therefore, the dynamic reward function for universities is $R(x)=(\text{1}-x)\zeta S+xP_{c}$, and the dynamic reward function for society is set as $R(y)=(\text{1}-y)\lambda S+yP_{s}$. The dynamic penalty function for universities is set as W(x)=(1-x)W, and the dynamic penalty function for society is set as $R(x)=(\text{1}-x)\zeta S+xP_{c}$.

This adaptive governance model has achieved precise intervention and a balance of rights and responsibilities by gradually imposing sanctions on universities that have not made sufficient progress in psychological education and by implementing hierarchical constraints on social institutions with persistently low resource openness. This fully dynamic mechanism, through dual regulation of rewards and punishments, builds an incentive-compatible ecosystem: the government enhances the efficiency of public fund utilization, and universities achieve the unification of their educational mission and collaborative behavior.

Choose the cooperation colleges and universities expected return for:


$$\pi_{x}=y\left(\xi R^{e}-C_{A}^{S}+E+(\text{1}-x)\zeta S+{xp}_{c}\right)+(\text{1}-y)\left(-G_{\text{2}}-C_{A}^{S}+(\text{1}-x)\zeta S+xp_{c}\right)$$

chose not to cooperation of colleges and universities expected return for:


$$\pi_{\text{1}-x}=y\left(E-L_{b}-(\text{1}-x)W)+(\text{1}-y)(-L_{b}-G_{\text{2}}-(\text{1}-x)W\right)$$

college replicated dynamic equation is as follows:


$$\frac{dx}{dt}=-x(x-\text{1})(L_{b}-C_{A}^{S}+W+S\zeta -Wx+p_{c}x+R^{e}\xi y-Sx\zeta )$$

The expected benefits of the social groups that choose to cooperate are:


$$\pi_{y}=x((\text{1}-\xi )R^{e}-C_{O}^{C}+\text{D}+(\text{1}-\text{y})\lambda S+{yP}_{s})+(\text{1}-x)\left(D-C_{O}^{C}+(\text{1}-\text{y})\lambda S+{yP}_{s}\right)$$

expected revenue of different social groups to choose not to cooperation:


$$\pi_{\text{1}-y}=x\left(-M-(\text{1}-y)F_{\text{1}}-(\text{1}-y)F_{\text{2}}\right)+(\text{1}-x)\left(-M-(\text{1}-y)F_{\text{1}}-(\text{1}-y)F_{\text{2}}\right)$$

social replicated dynamic equation is as follows:


$$\frac{dy}{dt}=-y(y-\text{1})(D-C_{O}^{C}+F_{\text{1}}+F_{\text{2}}+M-F_{\text{1}}y-F_{\text{2}}y+S\lambda +R^{e}x+P_{s}\text{y}-S\lambda y-R^{e}x\xi )$$

system evolution can be drawn from a total of five replication dynamic equilibrium as (0, 0), (0, 1), (1, 0), (1, 1), $\left(x^{*},y^{*}\right)$. Among them $\left(x^{*},y^{*}\right)=(-\frac{C_{O}^{C}-D-M-P_{s}\text{y}+F_{\text{1}}(y-\text{1}){+F}_{\text{2}}(y-\text{1})S\lambda (y-\text{1})}{R^{e}(\xi -\text{1})},\frac{C_{A}^{S}-L_{b}-p_{c}x-W(x-\text{1})+S\zeta (x-\text{1})}{R^{e}\xi })$ in X*∈[0,1],Y*∈[0,1], the local stability analysis method based on jacobian matrix, the system of five equilibrium stability analysis, the results are shown in Table 10.

**Table 10:** Local Stability of the Equilibrium Point.

Equilibrium Point	Sign of Tr(J)	Det(J) Sign	Result
(0,0)	+	–	ESS
(0,1)	+	+	Unstable
(1,0)	N	–	Saddle point
(1,1)	N	–	Saddle Point

**Fig 22:**
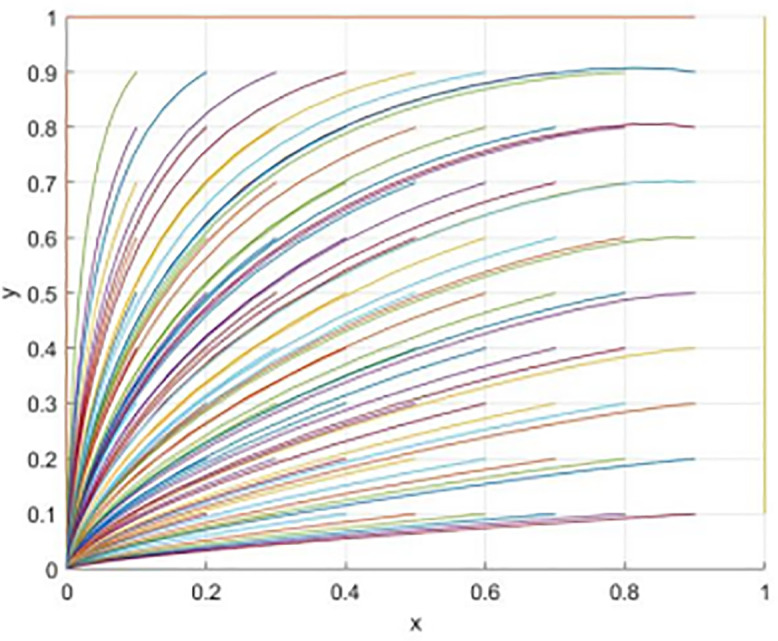
Evolutionary processes under a dual-dynamic mechanism.

**Fig 23:**
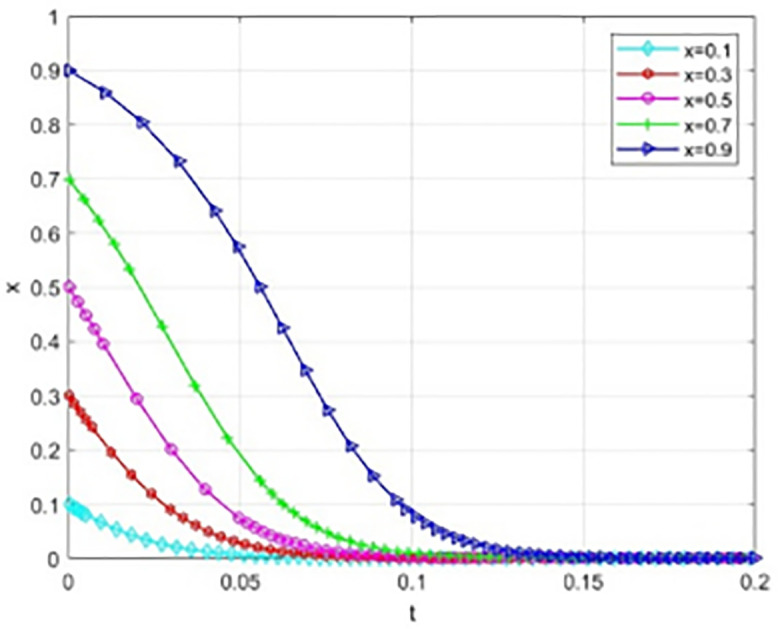
The impact of initial social values on evolutionary outcomes.

**Fig 24:**
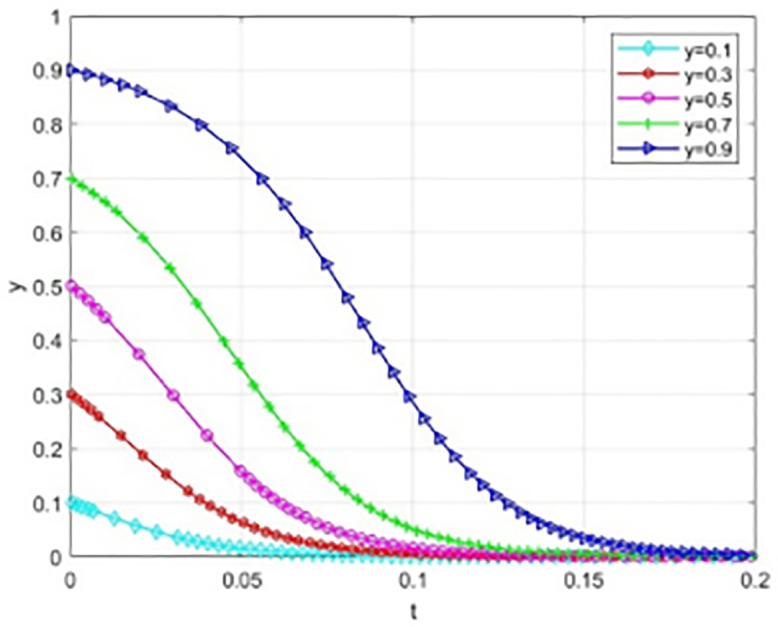
The impact of 22initial values in higher.

The following simulation is used to prove that the parameter Settings of the above results remain unchanged (Figs 22–24).

## 5. Conclusion

Evolutionary game theory is used to develop an analysis framework that can deal with both the static and the dynamic case where the government intervenes. This framework outputs the workings of the collaboration system, the balances and ways to improve the incentivized system. Theoretical basis and operational paths for breaking the deadlock of cooperation and building a sustainable education ecological system. The results suggest that traditional static intervention can help at the beginning of cooperation, but in the long term it will rely on government financial subsidies and be unable to achieve self-sustaining coordinated development. By adopting a dynamic incentive mechanism based on “performance grants and reputation incentives”, a combined model of dynamic rewards and static punishments is formed as the optimal choice. This way can guide the system move toward the steady-state and balanced direction. When the subsidies are bound, the promotion of the system evolution occurs via a distribution of 5:2. When subsidies are sufficient, a near 5:3 balance is more favorable for achieving a good and efficient synergy. In general, although dynamic subsidies should be inclined towards universities, it is necessary to prevent excessive imbalance.

It should be noted that although the dual dynamic model is theoretically excellent, this study found that the dynamic reward of “performance grant - reputation incentive” failed to achieve the expected results within the dual dynamic framework. Therefore, it is suggested that the government take a “dynamic rewards + static penalties” hybrid model when perfecting the collaborative incentive mechanism. Link the financial and reputation incentives with the actual performance, make it clear what is the cost of acting: no act, the system cannot be stable, then it is possible to reach both the improvement of incentives and the system stability.

From the perspective of economic investment and long-term benefit incentives, the government’s funding of a collaborative system for social psychological education in colleges and universities is a high-return strategic investment. Driven by the linkage mechanism of “performance grant - reputation incentive”, universities have been involved in the construction of collaborative education to improve their reputation. Improved reputations directly attract more prospective students and social attention, creating **a virtuous cycle connecting reputation, resource acquisition, and educational effectiveness.** As the quality of mental health education improves, graduates will have stronger psychological qualities and better social adaptability, providing high-quality human resources for society. This not only raises the overall quality of the labor market, but also promotes social harmony and economic development at a deeper level, creating **a positive feedback loop where the mental health of talents improves social productivity, fosters economic prosperity, and ultimately enhances national strength.** Practically, this study provides a feasible plan for the government to build a joint education governance system. Government is better to change it as a “rule maker, “ and a “fine performer”, turning behaviors like running together’s psychological course jointly, doing crisis together as well as sharing your resources for reputation gains. It also requires setting differentiated grant conversion coefficients and project priorities to form a positive cycle of “behavior - incentives - reputation - benefits”; At the same time, the government should optimize the cost-sharing and risk constraint mechanism, reduce cooperation costs through targeted subsidies, and enhance the reputational penalty and material constraint on inaction.

This study still has its own defects which later research can continue to explore on three aspects, that is, the occurrence of game predicaments is limited by the static form of strategy structures, but also restricted by the dynamic form of strategy distribution. Based on this, we will proceed to build multi-agent models. Second, in the dimension of the subject, future research should take multiple participants such as families and communities into account to construct a more complete multi-actor evolutionary game model. The third, in terms of the mechanism design section, one should focus on the progressive formulation of reputation incentives, multi-period dynamic optimization plans, and the impact of digital technology on the circulation of incentives. Finally, in terms of verification dimension, empirical study, questionnaires, questionnaires, case trace, to verify the effect of the mechanism in different Region and Type of university.

## Supporting information

S1 Appendix Supplementary Jacobian Matrix. (DOCX)

S1 File Code Simulation of Evolutionary Game Model. (DOCX)
