# Supplementary material for: Research on the path of social psychological collaborative education in colleges and universities driven by the dynamic reward and punishment mechanism of the government
Source: PLoS One. 2026 Feb 19;21(2):e0340411. doi: 10.1371/journal.pone.0340411 (PMC12919842; doi:10.1371/journal.pone.0340411)
Supplement: S1 Appendix — (DOCX) [file pone.0340411.s001.docx]

Appendix1

3.2.2 Jacobian matrix


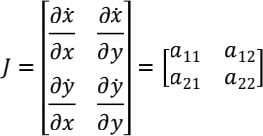


a*11* =
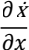
 = — (x — 1)(Lb — C + W + PC + Sζ + R *e*ξy) — x(Lb — C + W + PC + Sζ + R *e*ξy)


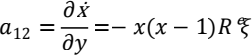


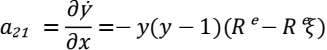


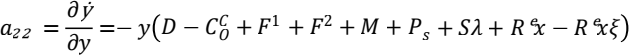


According to the criterion of the Evolutionarily Stable Strategy (ESS), if the following two conditions are met at the same time：

tr(J) = a*11* + a*zz* < 0


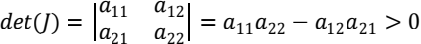


Appendix2

4.1The system Jacobian matrix is :


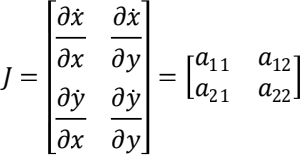


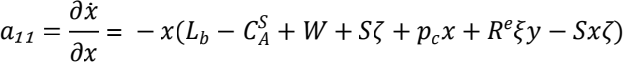


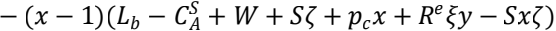


— x(x — 1)(pc — Sζ)


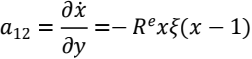


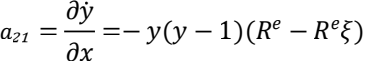


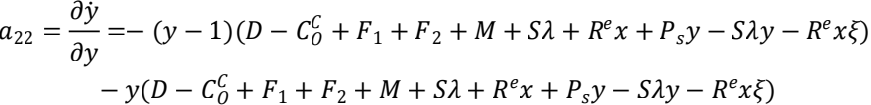


— Y(Y — 1)(PS — Sλ)

Appendix3

The system Jacobian matrix is

:


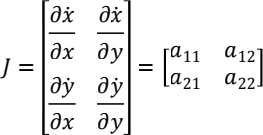


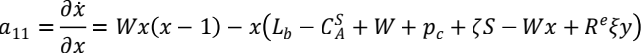


—(x — 1)(Lb — C + W + pC + Sζ — Wx + Re ξY)


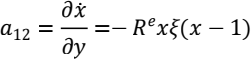


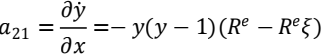


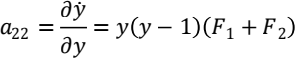


— (Y — 1)(D — C + F1 + F2 + M + PS — F1Y — F2Y + Sλ + Rex — Rexξ) — Y(D

— C + F1 + F2 + M + PS — F1Y — F2Y + Sλ + Rex — Rexξ)

Appendix4

The system Jacobian matrix is

:


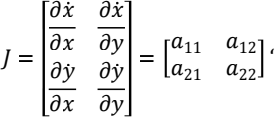


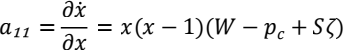


— (x — 1)(Lb — C + W + Sζ — Wx + pcx + Re ξy — Sxζ)

— x(Lb — C + W + Sζ — Wx + pcx + Re ξy — Sxζ)


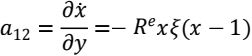


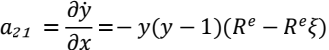


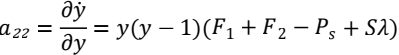


—(Y — 1)(D — C + F1 + F2 + M — F1Y — F2Y + Sλ + Rex + PSY — SλY — Rexξ)

—Y(D — C + F1 + F2 + M — F1Y — F2Y + Sλ + Rex + PSY — SλY — Rexξ)
