# Supplementary material for: Research on the path of social psychological collaborative education in colleges and universities driven by the dynamic reward and punishment mechanism of the government
Source: PLoS One. 2026 Feb 19;21(2):e0340411. doi: 10.1371/journal.pone.0340411 (PMC12919842; doi:10.1371/journal.pone.0340411)
Supplement: S1 File — (DOCX) [file pone.0340411.s002.docx]

function dydt = liangfang(t, y, xi, Cas, Re, Coc, D, Lb, M, W, pc, S, zeta, F1, F2, ps, lambda)

dydt=zeros(2,1);%%Two-dimensional first-order system of equations

dydt(1)=-y(1)*(y(1) - 1)*(Lb - Cas + W + pc + S*zeta + Re*xi*y(2));%xequation

dydt(2)=-y(2)*(y(2) - 1)*(D - Coc + F1 + F2 + M + ps + S*lambda + Re*y(1) - Re*y(1)*xi);%yequation

end

%Dynamic reward

function dydt = liangfang1(t, y, xi, Cas, Re, Coc, D, Lb, M, W, pc, S, zeta, F1, F2, ps, lambda)

dydt=zeros(2,1);%% Two-dimensional first-order system of equations

dydt(1)=-y(1)*(y(1) - 1)*(Lb - Cas + W + pc*y(1) + S*zeta + Re*xi*y(2)-S*y(1)*zeta);% Equation

dydt(2)=-y(2)*(y(2) - 1)*(D - Coc + F1 + F2 + M + ps*y(2) + S*lambda + Re*y(1) - Re*y(1)*xi-S*lambda*y(2));% Equationend

%Dynamic penalty

function dydt = liangfang2(t, y, xi, Cas, Re, Coc, D, Lb, M, W, pc, S, zeta, F1, F2, ps, lambda)

dydt=zeros(2,1);%% Two-dimensional first-order system of equations

dydt(1) = -y(1)*(y(1) - 1)*(Lb - Cas + W*(1-y(1)) + pc + S*zeta + Re*xi*y(2));

dydt(2) = -y(2)*(y(2) - 1)*(D - Coc + (F1 + F2)*(1-y(2)) + M + ps + S*lambda + Re*y(1) - Re*y(1)*xi);

end

%Dual dynamic

function dydt = liangfang4(t, y, xi, Cas, Re, Coc, D, Lb, M, W, pc, S, zeta, F1, F2, ps, lambda)

dydt=zeros(2,1);%% Two-dimensional first-order system of equations

dydt(1) = -y(1)*(y(1) - 1)*(Lb - Cas + W*(1-y(1)) + pc*y(1) + S*zeta*(1-y(1)) + Re*xi*y(2));

dydt(2) = -y(2)*(y(2) - 1)*(D - Coc + (F1 + F2)*(1-y(2)) + M + ps*y(2) + S*lambda*(1-y(2)) + Re*y(1) - Re*y(1)*xi);

end

clc;clear;%%Clear workspace and command window

%%%%1

xi = 0.5; Cas = 132; Re = 30; Coc = 102; D = 10; Lb = 50; M = 20; W =28; pc = 20; S = 10; zeta = 0.5; F1 = 8; F2 = 10; ps = 30; lambda = 0.5;%Parameter assignment

[t,y]=ode45(@(t,y) liangfang(t, y, xi, Cas, Re, Coc, D, Lb, M, W, pc, S, zeta, F1, F2, ps, lambda),[0,10],[0.9,0.9]); %%%Algorithm for solving the equation,equation running time(number of iterations),initial values of x and y

p=plot(t,y(:,2),'h-','linewidth',1,'markersize',5);

p.MarkerIndices = 1:1:length(y(:,2));%%%% Indicates the number of markers generated;the larger the middle 1,the larger the marker interval

hold on

%%%%1.5

xi = 0.5; Cas = 132; Re = 30; Coc = 102; D = 10; Lb = 50; M = 20; W =28; pc = 20; S = 15; zeta = 0.5; F1 = 8; F2 = 10; ps = 30; lambda = 0.5;% Parameter assignment

[t,y]=ode45(@(t,y) liangfang(t, y, xi, Cas, Re, Coc, D, Lb, M, W, pc, S, zeta, F1, F2, ps, lambda),[0,10],[0.9,0.9]); %%% Algorithm for solving the equation,equation running time(number of iterations),initial values of x and y

p=plot(t,y(:,2),'h-','linewidth',1,'markersize',5);

p.MarkerIndices = 1:1:length(y(:,2));%%%% Indicates the number of markers generated;the larger the middle 1,the larger the marker interval hold on

%%%%2

xi = 0.5; Cas = 132; Re = 30; Coc = 102; D = 10; Lb = 50; M = 20; W =28; pc = 20; S = 20; zeta = 0.5; F1 = 8; F2 = 10; ps = 30; lambda = 0.5;% Parameter assignment

[t,y]=ode45(@(t,y) liangfang(t, y, xi, Cas, Re, Coc, D, Lb, M, W, pc, S, zeta, F1, F2, ps, lambda),[0,10],[0.9,0.9]); %%% Algorithm for solving the equation,equation running time(number of iterations),initial values of x and y

p=plot(t,y(:,2),'s-','linewidth',1,'markersize',5);

p.MarkerIndices = 1:1:length(y(:,2));%%%% Indicates the number of markers generated;the larger the middle 1,the larger the marker interval hold on

%%%%3

xi = 0.5; Cas = 132; Re = 30; Coc = 102; D = 10; Lb = 50; M = 20; W =28; pc = 20; S = 30; zeta = 0.5; F1 = 8; F2 = 10; ps = 30; lambda = 0.5;% Parameter assignment

[t,y]=ode45(@(t,y) liangfang(t, y, xi, Cas, Re, Coc, D, Lb, M, W, pc, S, zeta, F1, F2, ps, lambda),[0,10],[0.9,0.9]); %%% Algorithm for solving the equation,equation running time(number of iterations),initial values of x and y

p=plot(t,y(:,2),'o-','linewidth',1,'markersize',5);

p.MarkerIndices = 1:1:length(y(:,2));%%%% Indicates the number of markers generated;the larger the middle 1,the larger the marker interval

hold on

%%%%4

xi = 0.5; Cas = 132; Re = 30; Coc = 102; D = 10; Lb = 50; M = 20; W =28; pc = 20; S = 40; zeta = 0.5; F1 = 8; F2 = 10; ps = 30; lambda = 0.5;% Parameter assignment [t,y]=ode45(@(t,y) liangfang(t, y, xi, Cas, Re, Coc, D, Lb, M, W, pc, S, zeta, F1, F2, ps, lambda),[0,10],[0.9,0.9]); %%% Algorithm for solving the equation,equation running time(number of iterations),initial values of x and y

p=plot(t,y(:,2),'d-','linewidth',1,'markersize',5);

p.MarkerIndices = 1:1:length(y(:,2));%%%% Indicates the number of markers generated;the larger the middle 1,the larger the marker interval hold on

grid on;

set(gca,'XTick',[0:0.2:2],'YTick',[0:0.1:1]) %%[0:axis unit length:1]

axis([0 2 0 1])%%Range of horizontal and vertical axes

xlabel('t');%%Name of horizontal axis

ylabel('y');%% Name of vertical axis

% ylabel('x');%% Name of vertical axis

legend('S=40', 'S=60', 'S=80', 'S=100','S=120'); %%%Legend

% axes('position',[0.2,0.2,0.3,0.3]); %Small figure tool for flexible use%The following is the detailed figure to be added

% axis([0.04 0.07 0.9 1])

xi = 0.5; Cas = 200; Re = 200; Coc = 190; D = 10; Lb = 70; M = 50; W =40; pc = 30; ps = 30; S =100; zeta = 0.5; F1 = 30; F2 = 20; lambda = 0.5;

[t,y]=ode45(@(t,y) liangfang1(t, y, xi, Cas, Re, Coc, D, Lb, M, W, pc, S, zeta, F1, F2, ps, lambda),[0,10],[0.4,0.4]); %%% Algorithm for solving the equation,equation running time(number of iterations),initial values of x and y p=plot(t,y(:,2),'h-','linewidth',1,'markersize',5);

p.MarkerIndices = 1:1:length(y(:,1));%%%% Indicates the number of markers generated;the larger the middle 1,the larger the marker interval hold on

%%%%2

xi = 0.5; Cas = 200; Re = 200; Coc = 190; D = 10; Lb = 70; M = 50; W =40; pc = 30; ps = 40; S =100; zeta = 0.5; F1 = 30; F2 = 20; lambda = 0.5;

[t,y]=ode45(@(t,y) liangfang1(t, y, xi, Cas, Re, Coc, D, Lb, M, W, pc, S, zeta, F1, F2, ps, lambda),[0,10],[0.4,0.4]); %%% Algorithm for solving the equation,equation running time(number of iterations),initial values of x and y

p=plot(t,y(:,2),'s-','linewidth',1,'markersize',5);

p.MarkerIndices = 1:1:length(y(:,1));%%%% Indicates the number of markers generated;the larger the middle 1,the larger the marker interval hold on

%%%%3

xi = 0.5; Cas = 200; Re = 200; Coc = 190; D = 10; Lb = 70; M = 50; W =40; pc = 30; ps = 50; S =100; zeta = 0.5; F1 = 30; F2 = 20; lambda = 0.5;

[t,y]=ode45(@(t,y) liangfang1(t, y, xi, Cas, Re, Coc, D, Lb, M, W, pc, S, zeta, F1, F2, ps, lambda),[0,10],[0.4,0.4]); %%% Algorithm for solving the equation,equation running time(number of iterations),initial values of x and y

p=plot(t,y(:,2),'o-','linewidth',1,'markersize',5);

p.MarkerIndices = 1:1:length(y(:,1));%%%% Indicates the number of markers generated;the larger the middle 1,the larger the marker interval hold on

grid on;

set(gca,'XTick',[0:0.05:2],'YTick',[0:0.1:1]) %%[0: axis unit length:1]

axis([0 0.4 0 1])%% Range of horizontal and vertical axes

xlabel('t');%% Name of horizontal axis

ylabel('y');%% Name of vertical axis

legend('ps=30', 'ps=40', 'ps=50'); %%%Legend

% axes('position',[0.2,0.2,0.3,0.3]); % Small figure tool for flexible use%The following is the detailed figure to be added

% axis([0.04 0.07 0.9 1])

values = 0.1:0.1:0.9;

colors = [

0.00, 0.45, 0.74; % Blue

0.85, 0.33, 0.10; % Orange

0.93, 0.69, 0.13; % Yellow

0.49, 0.18, 0.56; % Purple

0.47, 0.67, 0.19; % Green

0.30, 0.75, 0.93; % Light blue

0.64, 0.08, 0.18; % Red

0.50, 0.50, 0.50; % Gray

0.60, 0.40, 0.80 % Black

];

color2 = [

0.00, 0.45, 0.74; % Blue

0.85, 0.33, 0.10; % Orange

0.93, 0.69, 0.13; % Yellow

0.49, 0.18, 0.56; % Purple

0.30, 0.75, 0.93; % Light blue

0.64, 0.08, 0.18; % Red

0.50, 0.50, 0.50; % Gray

0.47, 0.67, 0.19; % Green

1.00, 0.50, 0.00; % Orange red

0.75, 0.00, 0.75; % Magenta

0.00, 0.75, 0.75; % Cyan

0.75, 0.75, 0.00; % Golden yellow

0.25, 0.25, 0.25; % Dark gray

0.90, 0.60, 0.80; % Pink

0.90, 0.50, 0.30

];

styles = {'-', '--', ':', '-.'};

marker = {'o', '+', '*', '.', 'x', 's', 'd', '^', 'v'};

% Generate all conbinations

[i_grid, j_grid] = meshgrid(values, values);

combinations = [i_grid(:), j_grid(:)];

% Calculate and sort

sums = combinations(:,1) + combinations(:,2);

[~, sort_idx] = sort(sums);

sorted_combinations = combinations(sort_idx, :);

legends={};

A = 0;

B = 0;

previous_sum_rounded = -1;

xi = 0.5; Cas = 210; Re = 200; Coc = 190; D = 14; Lb = 70; M = 50; W =40; pc = 60; ps = 30; S =90; F1 = 30; F2 = 20;

% Traverse

figure(2)

for k = 1:size(sorted_combinations, 1)

i = sorted_combinations(k, 1);

j = sorted_combinations(k, 2);

current_sum = i + j;

B = B+1;

% Check if the integer part of i+j changes

current_sum_rounded = round(current_sum * 10) / 10;

% Check if the rounded value changes

if current_sum_rounded ~= previous_sum_rounded

A = A + 1;

previous_sum_rounded = current_sum_rounded;

% pause(1)

% figure(A)

end

% if A>7

%

% break

% end

%

% if A< 7

% B=0;

%

% continue

% end

fprintf('Step counter increment: Current step = %d, i+j = %.1f \n', A, current_sum);

color_idx = mod(A-1, size(colors, 1)) + 1;

zeta = i; lambda = j;

grid on%%%Grid

axis([0 1 0 1 0 0.5])%%% Range of horizontal and vertical axes

xlabel('x');% Name of horizontal axis

ylabel('y');% Name of vertical axis

zlabel("t")

set(gca,"XTick",[0:0.5:1],"YTick",[0:0.5:1])

[t,y]=ode45(@(t,y) liangfang1(t, y, xi, Cas, Re, Coc, D, Lb, M, W, pc, S, zeta, F1, F2, ps, lambda),[0,10],[0.5,0.5]); %%% Algorithm for solving the equation,equation running time(number of iterations),initial values of x and y

% Global

plot3(y(:,1),y(:,2),t,'color',colors(color_idx,:),'linewidth',0.5);

%The following is with legend

% p=plot3(y(:,1),y(:,2),t,"color",color2(B,:),'linewidth',1);

% p.DisplayName = ['ζ=', num2str(i),'λ=', num2str(j)];

hold on

axis([0 1 0 1 0 0.4])

end

% legend('show')
